# Supplementary material for: Financial and regulatory interventions to reduce unnecessary caesarean sections: An updated scoping review
Source: PLOS Glob Public Health. 2026 Feb 9;6(2):e0005830. doi: 10.1371/journal.pgph.0005830 (PMC12885279; doi:10.1371/journal.pgph.0005830)
Supplement: S2 Appendix — (DOCX) [file pgph.0005830.s002.docx]

**S2 Appendix. Type of interventions, mapped to the WHO health system building blocks.**

| WHO health system building blocks | Definition of interventions | Example of interventions |
| --- | --- | --- |
| Leadership & Governance | Any implementation of policy, regulation, and legislation, including initiatives to strengthen the role of patients or communities in health governance, designed to reduce the unnecessary use of CS. | Laws and regulations promoting setting CS rates |
|  |  | Policies limiting financial and legal liability in litigation cases |
|  |  | Promotion of vaginal birth (demand-side initiatives) |
|  |  | Polices on patient safety plans including strengthening role for patients and communities in monitoring quality of care towards improving health facility/ provider governance and accountability |
| Financing | Any implementation of strategy or reform in healthcare financing, including contractual arrangements for health workers and facilities and payment systems designed to influence service delivery and reduce unnecessary CS. | Including vs. excluding CS from benefit package |
|  |  | Changing conditions of access to CS services – e.g.  introduce or remove co-payments and user fees on CS), imposing a referral model |
|  |  | Shifting to more strategic provider selection – e.g. accreditation criteria more restrictive for CS services; removing vs. expanding coverage to private sector |
|  |  | Shifting to active contracting – i.e. establishing volume or CS rate targets |
|  |  | Changing payment methods for provision of CS services – e.g. Equalising payment rates for CS and vaginal births, providing higher payment for vaginal births than CS, |
|  |  | Blending payment methods through the introduction of performance add-ons e.g. applying financial rewards or penalties in case of respect or failure to respect volume of CS ratio targets, e.g. setting a volume cap from which a lower-case payment applies, pay-for-performance model ( CS rate as part of the P4P indicators) |
| Health workforce | Any strategy, implementation, or reform focused on the training and education of the health workforce | Educational interventions to improve adherence to evidence-based clinical practices |
|  |  | Audit, feedback, and peer review to ensure compliance with evidence-based protocols and guidelines |
|  |  | Requiring a second opinion for CS indications |
| Service delivery | Any approach or reform in the organization of service delivery aimed at reducing unnecessary CS | Collaborative midwifery-obstetrician or midwifery-led model of care |
|  |  | Modifying the physical and sensory environment of labor and delivery |
|  |  | Establishing setting-specific goals for CS rates |
|  |  | Strategies to transform organizational culture |
| Health information systems | Any interventions leveraging health information systems to reduce unnecessary CS use | Publication and benchmarking of CS rates |
|  |  | Health information systems to track CS rates |
|  |  | Health information system to enable data-driven decision-making for mode of birth |
|  |  | Systematic implementation of the Robson classification |
| Access to essential medicines | Any approach or intervention to ensure necessary medications and surgical supplies are available and affordable to support alternative interventions and management strategies that might prevent the need for unnecessary CS | Improving access to assisted vaginal birth instruments (vacuum extractor, forceps, suture materials, etc) |
|  |  | Improving access to induction and augmentation of labor |
|  |  | Improving access of pain management during labour |
