# Supplementary material for: Financial and regulatory interventions to reduce unnecessary caesarean sections: An updated scoping review
Source: PLOS Glob Public Health. 2026 Feb 9;6(2):e0005830. doi: 10.1371/journal.pgph.0005830 (PMC12885279; doi:10.1371/journal.pgph.0005830)
Supplement: S3 Appendix — (DOCX) [file pgph.0005830.s003.docx]

**S3 Appendix. Search strategy**

| MEDLINE PubMed | | |
| --- | --- | --- |
| Concept | **Search string** | **Results** |
| #1 | "Natural Childbirth"[Mesh] OR "Vaginal Birth after Cesarean"[Mesh] OR "Cesarean Section"[Mesh] OR "normal childbirth" OR "normal birth" OR "vaginal birth" OR "vaginal childbirth" OR “Abdominal Deliveries”[TW] OR “C Section” [TW] OR “C Sections” [TW] OR Postcesarean[TW] OR Post CS [TW] OR Cesarean[TW] OR CS [TW] | 97,242 |
| #2 | "Legislation" [Publication Type] OR "Legislation as Topic"[Mesh] OR "Legislation, Medical"[Mesh] OR  "Taxes”[MeSH] OR  "Social Control, Formal"[Mesh] OR  "Legal Case" [Publication Type] OR  "legislation  and  jurisprudence" [Subheading] OR “Fiscal Policy”[MeSH] OR “economics”[subheading] OR laws [TW]  OR law[TW] OR legislation[TW]  OR jurisprudence[TW]  OR legal [TW] OR tax[tiab] OR taxes[tiab] OR taxation[tiab] OR taxed[tiab] OR taxing[tiab] OR subsidy[tiab] OR subsidies[tiab] OR subsidized[tiab] OR subsidised [TIAB] OR incentive[tiab] OR incentives[tiab] OR discount[tiab] OR discounts[tiab] OR discounted[tiab] OR price[tiab] OR prices[tiab] OR priced[tiab] OR  fiscal [tiab] OR rebate[tiab] OR vouchers[tiab] OR rebates[tiab] OR levy[tiab] OR financial [TIAB] | 1,503,030 |
| #3 | #1 AND #2 | 3,720 |
| #4-date filter | Filter to 2019-2024 | 628 |
| #5-Human Filter | NOT ("animals"[MeSH] NOT "humans"[MeSH]) AND "humans"[MeSH] | 548 |

| EMBASE Ovid | | |
| --- | --- | --- |
| Concept | **Search string** | **Results** |
| #1 | exp Natural Childbirth/ OR exp Vaginal Delivery/ OR exp Cesarean Section/ OR exp Vaginal Birth after Cesarean/ OR ("Abdominal Deliveries" OR "C section" OR "C sections" OR Postcesarean OR Post CS OR Cesarean OR CS OR "natural childbirth" OR "normal childbirth" OR "normal birth" OR "vaginal birth" OR "vaginal childbirth" OR "vaginal delivery").ti,ab,kw. | 195,659 |
| #2 | exp Law/ OR exp Medicolegal Aspect/ OR exp Tax/ OR exp Social Control/ OR exp Fiscal Policy/ OR (laws OR law OR legislation OR jurisprudence OR legal).ti,ab,kw OR (tax OR taxes OR taxation OR taxed OR taxing OR subsidy OR subsidies OR subsidized OR subsidised OR incentive OR incentives OR discount OR discounts OR discounted OR price OR prices OR priced OR fiscal OR rebate OR vouchers OR rebates OR levy OR financial).ti,ab,kw | 1,213,658 |
| #3 | #1 AND #2 | 5588 |
| #4-date filter | Filter to 2019-2024 | 1822 |
| #5-Human Filter | Filter to Human | 1799 |

| CINAHL | | |
| --- | --- | --- |
| Concept | **Search string** | **Results** |
| #1 | MH "Childbirth+" OR MH "Vaginal Birth After Cesarean" OR MH "Cesarean Section+" OR MH "Vaginal Birth+" OR TI  Cesarean OR TI  CS OR  TI (C-sections) OR  TI(C-section) OR TI (Abdominal Deliveries) OR TI (Abdominal Delivery) OR TI Postcesarean OR TI Post CS OR TI (Post cesarean)  OR TI (Post CS ) OR AB  Cesarean OR AB  CS OR  AB (C-sections) OR  AB (C-section) OR AB (Abdominal Deliveries) OR AB (Abdominal Delivery) OR AB Postcesarean OR AB Post CS OR AB (Post cesarean)  OR AB (Post  CS ) | 65,595 |
| #2 | MH "Legislation+" OR MH "Legislation, Medical+" OR MH "Taxes+" OR MH "Social Control+" OR laws OR law OR legislation OR jurisprudence OR legal OR TI tax OR AB tax OR TI taxes OR AB taxes OR TI taxation OR AB taxation OR TI taxed OR AB taxed OR TI taxing OR AB taxing OR TI subsidy OR AB subsidy OR TI subsidies OR AB subsidies OR TI subsidized OR AB subsidized OR TI subsidised OR AB subsidised OR TI incentive OR AB incentive OR TI incentives OR AB incentives OR TI discount OR AB discount OR TI discounts OR AB discounts OR TI discounted OR AB discounted OR TI price OR AB price OR TI prices OR AB prices OR TI priced OR AB priced OR TI fiscal OR AB fiscal OR TI rebate OR AB rebate OR TI vouchers OR AB vouchers OR TI rebates OR AB rebates OR TI levy OR AB levy OR TI financial OR AB financial | 641,766 |
| #3 | #1 AND #2 | 4083 |
| #4-date filter | Filter to 2019-2024 | 1047 |

| GIM (Global Health Library), <http://www.globalhealthlibrary.net> | | |
| --- | --- | --- |
| Concept | **Search string** | **Results** |
| #1 | ((Cesarean ) OR ( CS ) OR (Cesareans ) OR ( CS s ) OR  (C-sections) OR (C-section) OR (Abdominal Deliveries) OR (Abdominal Delivery) OR (Postcesarean)  OR (Post CS )) | 15,404 |
| #2 | ((laws) OR (law) OR (legislation) OR (jurisprudence) OR (legal) OR (tax) OR (taxes) OR (taxation) OR (taxed) OR (taxing) OR (subsidy) OR (subsidies) OR (subsidized) OR (subsidised) OR (incentive) OR (incentives) OR (discount) OR (discounts) OR (discounted) OR (price) OR (prices) OR (priced) OR (fiscal) OR (rebate) OR (vouchers) OR (rebates) OR (levy) OR (financial)) | 79,515 |
| #3 - #1 AND #2 | ((Cesarean ) OR ( CS ) OR (Cesareans ) OR ( CS s ) OR  (C-sections) OR (C-section) OR (Abdominal Deliveries) OR (Abdominal Delivery) OR (Postcesarean)  OR (Post CS ))  AND ((laws) OR (law) OR (legislation) OR (jurisprudence) OR (legal) OR (tax) OR (taxes) OR (taxation) OR (taxed) OR (taxing) OR (subsidy) OR (subsidies) OR (subsidized) OR (subsidised) OR (incentive) OR (incentives) OR (discount) OR (discounts) OR (discounted) OR (price) OR (prices) OR (priced) OR (fiscal) OR (rebate) OR (vouchers) OR (rebates) OR (levy) OR (financial)) | 294 |
| #4-date filter | Filter to 2019-2024 | 61 |

| Ebsco MultiDisciplinary Databases | | |
| --- | --- | --- |
| Concept | **Search string** | **Results** |
| #1 | (SU "Vaginal Birth After Cesarean" OR SU "Cesarean Section" OR SU "Vaginal Birth" OR TI  Cesarean OR TI  CS OR  TI “C-sections” OR  TI “C-section” OR TI “Abdominal Deliveries” OR TI “Abdominal Delivery” OR TI Postcesarean OR TI Post CS OR TI “Post cesarean”  OR TI “Post CS ”) AND (SU "Legislation" OR SU "Legislation, Medical" OR SU "Taxes" OR SU "Social Control" OR laws OR law OR legislation OR jurisprudence OR legal OR TI tax OR AB tax OR TI taxes OR AB taxes OR TI taxation OR AB taxation OR TI taxed OR AB taxed OR TI taxing OR AB taxing OR TI subsidy OR AB subsidy OR TI subsidies OR AB subsidies OR TI subsidized OR AB subsidized OR TI subsidised OR AB subsidised OR TI incentive OR AB incentive OR TI incentives OR AB incentives OR TI discount OR AB discount OR TI discounts OR AB discounts OR TI discounted OR AB discounted OR TI price OR AB price OR TI prices OR AB prices OR TI priced OR AB priced OR TI fiscal OR AB fiscal OR TI rebate OR AB rebate OR TI vouchers OR AB vouchers OR TI rebates OR AB rebates OR TI levy OR AB levy OR TI financial OR AB financial) AND filter date 2019-2024 | 233 |
