# Supplementary material for: Financial and regulatory interventions to reduce unnecessary caesarean sections: An updated scoping review
Source: PLOS Glob Public Health. 2026 Feb 9;6(2):e0005830. doi: 10.1371/journal.pgph.0005830 (PMC12885279; doi:10.1371/journal.pgph.0005830)
Supplement: S5 Appendix — (DOCX) [file pgph.0005830.s005.docx]

**S5 Appendix. Critical appraisal.**

| Author (year) | Risk of bias | | | | | | | Certainty assessment | | | | | Certainty |
| --- | --- | --- | --- | --- | --- | --- | --- | --- | --- | --- | --- | --- | --- |
|  | **Confounding** | **Selection of participants into the study** | **Classification of interventions** | **Deviations from intended interventions** | **Missing data** | **Measurement of outcomes** | **Selection of the reported result** | **Overall risk of bias** | **Inconsistency** | **Indirectness** | **Imprecision** | **Other aspects** |  |
| Barili 2021 | Low | Moderate | Low | Low | Low | Low | Low | Not serious | Single study | Not serious | Not serious | None | Low |
| Behzadifar 2020 | Moderate | Serious | Low | Moderate | Moderate | Moderate | Low | Serious | Single study | Not serious | Not serious | None | Very low |
| Lotfi 2021 | Moderate | Low | Low | Moderate | Moderate | Moderate | Low | Serious | Single study | Not serious | Not serious | None | Very low |
| Pilvar 2021 | Low | Low | Low | Low | Moderate | Moderate | Low | Not serious | Single study | Not serious | Not serious | None | Low |
| Mosaddeq 2020 | Low | Low | Low | Moderate | Moderate | Moderate | Low | Not serious | Single study | Not serious | Not serious | None | Low |
| Parwanehsadeghi 2018 | Serious | Low | Low | Moderate | Serious | Moderate | Low | Serious | Single study | Not serious | Not serious | None | Very low |
| Rashidian 2019 | Moderate | Low | Low | Low | Moderate | Moderate | Low | Serious | Single study | Not serious | Not serious | None | Low |
| Cozzi-Glaser 2024 | Low | Low | Low | Low | Moderate | Moderate | Low | Not serious | Single study | Not serious | Not serious | None | Low |
| Meng 2019 | Low | Low | Low | Low | Moderate | Moderate | Low | Not serious | Single study | Not serious | Not serious | None | Low |
| Nedberg 2022 | Low | Low | Low | Low | Moderate | Moderate | Low | Not serious | Single study | Not serious | Not serious | None | Low |
| Park 2022 | Low | Low | Low | Low | Moderate | Moderate | Low | Not serious | Single study | Not serious | Not serious | None | Low |
| Rosenstein 2021 | Low | Low | Low | Moderate | Low | Moderate | Low | Serious | Single study | Not serious | Not serious | None | Low |
| Safrin 2023 | Critical | Moderate | Low | Moderate | Moderate | Moderate | Low | Very serious | Single study | Not serious | Serious | None | Very low |
| Snowden 2020 | Low | Low | Low | Moderate | Moderate | Moderate | Low | Serious | Single study | Not serious | Not serious | None | Low |
| Escuriet-Peiro 2015 | Critical | Low | Low | Moderate | Moderate | Moderate | Low | Very serious | Single study | Not serious | Not serious | None | Very low |
| SukruBudak 2020 | Critical | Low | Low | Moderate | No information | Moderate | Low | Very serious | Single study | Not serious | Serious | None | Very low |
| Lo 2008 | Moderate | Serious | Low | Low | Low | Moderate | Low | Serious | Single study | Not serious | Not serious | None | Very low |
| Keeler 1996 | Moderate | Serious | Low | Low | Low | Moderate | Low | Serious | Single study | Not serious | Not serious | None | Very low |
| Liu 2007 | Moderate | Low | Low | Low | Low | Moderate | Low | Not serious | Single study | Not serious | Not serious | None | Low |
| Kim 2016 | Moderate | Low | Low | Low | Low | Moderate | Low | Not serious | Single study | Not serious | Not serious | None | Low |
| Lee 2007 | Moderate | Low | Low | Low | Low | Moderate | Low | Not serious | Single study | Not serious | Not serious | None | Low |
| Chen 2014 | Moderate | Low | Low | Low | Low | Moderate | Low | Not serious | Single study | Not serious | Not serious | None | Low |
| Kozhimannil 2018 | Moderate | Low | Low | Low | Low | Moderate | Low | Not serious | Single study | Not serious | Not serious | None | Low |
| Liu 2013 | Moderate | Low | Low | Low | Low | Moderate | Low | Not serious | Single study | Not serious | Not serious | None | Low |
| Tsai 2006 | Moderate | Moderate | Low | Low | Low | Moderate | Low | Serious | Single study | Not serious | Not serious | None | Very low |
| Misra 2008 | Moderate | Low | Low | Low | Low | Moderate | Low | Not serious | Single study | Not serious | Not serious | None | Low |
| Chen 2016 | Moderate | Moderate | Low | Low | Moderate | Moderate | Low | Serious | Single study | Not serious | Not serious | None | Very low |
| Karami 2018 | Low | Low | Low | Low | Moderate | Moderate | Low | Not serious | Single study | Not serious | Not serious | None | Low |
| Studnicki 1997 | Moderate | Low | Low | Low | Low | Moderate | Low | Not serious | Single study | Not serious | Not serious | None | Low |
| Yu 2017 | Moderate | Low | Low | Low | Low | Moderate | Low | Not serious | Single study | Not serious | Not serious | None | Low |
| Snowden 2016 | Moderate | Low | Low | Low | Low | Moderate | Low | Not serious | Single study | Not serious | Not serious | None | Low |
| Borem 2020 | Moderate | Low | Low | Low | Low | Moderate | Low | Not serious | Single study | Not serious | Not serious | None | Low |
