# Supplementary material for: Financial and regulatory interventions to reduce unnecessary caesarean sections: An updated scoping review
Source: PLOS Glob Public Health. 2026 Feb 9;6(2):e0005830. doi: 10.1371/journal.pgph.0005830 (PMC12885279; doi:10.1371/journal.pgph.0005830)
Supplement: S6 Appendix — (DOCX) [file pgph.0005830.s006.docx]

**S6 Appendix. Outcomes and impact reported for each study.**

| Author (year) | Outcome | Effect |
| --- | --- | --- |
| Chen 2014 | CS rate | Possible harm |
|  | Elective CS | Possible benefit |
| Liu 2013 | CS rate | Possible no difference of effect |
| Kozhimannil 2018 | CS rate | Possible benefit |
|  | Maternal morbidity (not specified) | Possible no difference of effect |
|  | Cost of birth | Possible benefit |
| Lee 2007 | CS rate | Possible no difference of effect |
| Kim 2016 | CS rate | Possible benefit |
| Chen 2016 | CS rate | No conclusion possible |
|  | CS medical expenditures | No conclusion possible |
| Misra 2008 | CS rate | Possible harm |
|  | VBAC | Possible benefit |
| Meng 2019 | CS rate | Possible benefit |
|  | Length of stay | Possible benefit |
|  | Readmissions/hospitalizations | Possible no difference of effect |
|  | Total spending per birth admission | Possible benefit |
|  | Government reimbursement expenses | Possible benefit |
|  | Out of pocket payments | Possible benefit |
|  | Medications costs | Possible benefit |
|  | Diagnostic testing costs | Possible benefit |
|  | Physician and therapeutic services costs | Possible benefit |
| Tsai 2006 | CS rate | No conclusion possible |
| Barili 2021 | CS rate | Possible benefit |
| Lo 2008 | CS rate | No conclusion possible |
|  | VBAC | No conclusion possible |
| Keeler 1996 | CS rate | No conclusion possible |
| Cozzi-Glaser 2024 | CS rate | Possible benefit |
|  | Birthweight | Possible benefit |
|  | Large-for-GA infants | Possible benefit |
|  | Neonatal hypoglycaemia | Possible benefit |
|  | NICU admissions | Possible no difference of effect |
| Snowden 2016 | CS rate | Possible benefit |
|  | Elective CS | Possible benefit |
|  | Induced labour | Possible no difference of effect |
|  | Stillbirth | Possible no difference of effect |
|  | Neonatal death | Possible no difference of effect |
|  | Neonatal seizures | Possible no difference of effect |
|  | NICU admissions | Possible no difference of effect |
|  | Assisted ventilation/respiratory distress syndrome | Possible no difference of effect |
| Studnicki 1997 | CS rate | Possible benefit |
| Escuriet-Peiro 2015 | CS rate | No conclusion possible |
|  | Assisted vaginal birth | No conclusion possible |
| Safrin 2023 | CS rate | No conclusion possible |
| Yu 2017 | CS rate | Possible benefit |
|  | Elective CS | Possible benefit |
| SukruBudak 2020 | CS rate | No conclusion possible |
|  | Neonatal mortality | Possible benefit |
| Nedberg 2022 | CS rate | Possible benefit |
|  | Assisted vaginal birth | Possible benefit |
|  | Induced labour | Possible benefit |
|  | NICU admissions | Possible no difference of effect |
|  | Neonatal mortality | Possible no difference of effect |
| Rosenstein 2021 | CS rate | Possible benefit |
| Park 2022 | CS rate | Possible harm |
| Liu 2007 | CS rate | Possible harm |
| Borem 2020 | CS rate | Possible benefit |
|  | CS by Robson group | Possible benefit |
|  | Vaginal birth | Possible benefit |
|  | Induced labour | Possible benefit |
|  | Maternal morbidity (not specified) | Possible no difference of effect |
|  | Maternal mortality | Possible benefit |
|  | NICU admissions | Possible no difference of effect |
|  | Neonatal mortality | Possible benefit |
|  | Obstetric anal sphincter injury | Possible benefit |
|  | Preterm birth | Possible benefit |
|  | Need for antibiotics other than routine prophylaxis | Possible benefit |
|  | Net promoter score | Possible no difference of effect |
|  | Women's experiences and perceptions of the interventions | Possible benefit |
| Karami Matin 2018 | CS rate | Possible harm |
| Behzadifar 2020 | CS rate | No conclusion possible |
| Lotfi 2021  Lotfi 2021 | CS rate | No conclusion possible |
|  | Vaginal birth | No conclusion possible |
| Pilvar 2021 | CS rate | Possible benefit |
| Mosaddeq 2020 | CS rate | Possible benefit |
| Parwanehsadeghi 2018 | CS rate | No conclusion possible |
|  | Vaginal birth | No conclusion possible |
|  | Readmissions/hospitalizations | No conclusion possible |
|  | Neonatal seizures | No conclusion possible |
|  | Assisted ventilation/respiratory distress syndrome | No conclusion possible |
|  | Chorioamnionitis/sepsis | No conclusion possible |
|  | Neonatal mortality | No conclusion possible |
| Rashidian 2019 | CS rate | Possible no difference of effect |
