# Supplementary material for: Financial and regulatory interventions to reduce unnecessary caesarean sections: An updated scoping review
Source: PLOS Glob Public Health. 2026 Feb 9;6(2):e0005830. doi: 10.1371/journal.pgph.0005830 (PMC12885279; doi:10.1371/journal.pgph.0005830)
Supplement: S7 Appendix — (DOCX) [file pgph.0005830.s007.docx]

**S7 Appendix. Table of included study characteristics.**

| Author (year) | Study design | Country | Income level | Health system level (implementation) | Year when study started | No of women/births involved | No of health facilities involved | List of interventions implemented | Elements of interventions | Complexity of interventions | Health system building blocks | CS baseline | Impact on CS |
| --- | --- | --- | --- | --- | --- | --- | --- | --- | --- | --- | --- | --- | --- |
| Chen 2014 | Retrospective before and after study | China (Taiwan) | High income country | National level | 2000 to 2010 | more than 1,000,000 | Not reported | Global budget payment (GBP) system, equalising fees by increasing VB fee to match CS, co-payment by women when CS not indicated | Benefit design, payment method | Complex financing intervention | Financing | 30-40% | Increase |
| Liu 2013 | Interrupted time series study | China (Taiwan) | High income country | Facility level | 2000 to 2010 | 10,000 to 100,000 | less than 5 | Global budget payment (GBP) system, post-CS peer reviews, and audit and feedback | Payment method, contracting including reporting obligations | Complex financing intervention | Financing | 30-40% | No change |
| Kozhimannil 2018 | Interrupted time series study | United States of America | High income country | Regional/state/provincial level | 2000 to 2010 | 100,000 to 1,000,000 | Not reported | Global budget payment (GBP) system | Payment method | Simple financing intervention | Financing | 20-30% | Reduce |
| Lee 2007 | Retrospective cohort study | Republic of Korea (South Korea) | High income country | National level | 2000 to 2010 | 100,000 to 1,000,000 | more than 100 | Diagnosis-related group (DRG) payment system | Two components of payment method (changes to DRG, changes from retrospective to prospective payment system) | Complex financing intervention | Financing | 40-50% | No change |
| Kim 2016 | Retrospective before and after study | Republic of Korea (South Korea) | High income country | National level | 2011 to 2021 | more than 1,000,000 | more than 100 | Diagnosis-related group (DRG) payment system | Two components of payment method (changes to DRG, changes from retrospective to prospective payment system) | Complex financing intervention | Financing | 30-40% | Reduce |
| Chen 2016 | Retrospective before and after study | China | High income country | Regional/state/provincial level | 2000 to 2010 | 1000 to 10,000 | less than 5 | Capitation based payment system | Benefit design, payment method | Complex financing intervention | Financing | 40-50% | Increase |
| Misra 2008 | Retrospective before and after study | United States of America | High income country | Regional/state/provincial level | Before 2000 | 100,000 to 1,000,000 | Not reported | Capitation based payment system | Provider selection, payment method | Complex financing intervention | Financing | 20-30% | Increase |
| Meng 2019 | Difference in Differences (DID) analysis | China | High income country | Regional/state/provincial level | 2000 to 2010 | 10,000 to 100,000 | Not reported | Episode-based bundled payment (EBP) | Payment method | Simple financing intervention | Financing | 30-40% | Reduce |
| Tsai 2006 | Retrospective before and after study | China (Taiwan) | High income country | National level | Before 2000 | 1000 to 10,000 | less than 5 | Case-based payment system | Payment method, benefit design | Complex financing intervention | Financing | Not clear | Reduce |
| Barili 2021 | Retrospective before and after study | Italy | High income country | Regional/state/provincial level | 2000 to 2010 | 100,000 to 1,000,000 | Not reported | Equalising fees by increasing VB fee to match CS + DRG | Payment method | Simple financing intervention | Financing | 20-30% | Reduce |
| Lo 2008 | Retrospective before and after study | China (Taiwan) | High income country | National level | 2000 to 2010 | more than 1,000,000 | Not reported | Equalising fees by increasing VB fee to match CS | Payment method | Simple financing intervention | Financing | 30-40% | No change |
| Keeler 1996 | Retrospective before and after study | United States of America | High income country | Regional/state/provincial level | Not reported | 10,000 to 100,000 | Not reported | Equalising fees by increasing VB fee and reducing CS fee | Payment method | Simple financing intervention | Leadership & Governance, Service Delivery | 20-30% | Reduce |
| Cozzi-Glaser 2024 | Retrospective cohort study | United States of America | High income country | Facility level | 2011 to 2021 | 10,000 to 100,000 | Not reported | Policy limiting elective induction/CS <39 weeks | Improvement of quality care | Simple regulatory intervention | Leadership & Governance | Less than 20% | Reduce |
| Snowden 2016 | Retrospective cohort study | United States of America | High income country | Regional/state/provincial level | 2011 to 2021 | 100,000 to 1,000,000 | Not reported | Policy limiting elective induction/CS <39 weeks | Formulating policy and strategic plans | Simple regulatory intervention | Leadership & Governance | Less than 20% | Reduce |
| Studnicki 1997 | Retrospective before and after study | United States of America | High income country | Regional/state/provincial level | Before 2000 | 100,000 to 1,000,000 | Not reported | Legislatively imposed practice guidelines | Formulating policy and strategic plans, ensuring accountability | Complex regulatory intervention | Financing, Leadership & Governance, Health Workforce, Health Service Delivery | Less than 20% | Reduce |
| Escuriet-Peiro 2015 | Descriptive study | Spain | High income country | Regional/state/provincial level | 2000 to 2010 | 100,000 to 1,000,000 | 10 to 50 | Strategy paper on accreditation, funding for infrastructure improvement, provider training, and women’s involvement in decision making | Formulating policy and strategic plans, improving of quality of care, putting in place lever tools for implementing policy | Complex regulatory intervention | Leadership & governance | 20-30% | Increase |
| Safrin 2023 | Descriptive study | United States of America | High income country | Regional/state/provincial level | 2000 to 2010 | more than 1,000,000 | Not reported | Damage caps implementation | Formulating policy and strategic plans | Simple regulatory intervention | Leadership & governance, service delivery, health workforce, health information system, financing | 30-40% | Increase |
| Yu 2017 | Retrospective before and after study | China | Upper middle income country | National level | 2000 to 2010 | 100,000 to 1,000,000 | less than 5 | Multifaceted interventions targeting women (health education, IEC dissemination, specific informed consent for CS based on maternal request) and providers (training, CS indications and guidelines dissemination, audit and feedback, painless vaginal birth, doula implementation), and policy implementation where CS rate monitored by Ministry of Health and penalties for providers if CS rate exceeds reasonable range | \| Formulating policy and strategic plans, ensuring accountability, putting in place levers or tools for implementing policy, improvement of quality of care \| \| --- \| | Complex regulatory intervention | Financing | More than 50% | Reduce |
| SukruBudak 2020 | Retrospective before and after study | Turkiye | Upper middle income country | National level | 2000 to 2010 | 100,000 to 1,000,000 | less than 5 | CS rates ceiling and penalty for providers exceeding reasonable CS rates | Formulating policy and strategic plans, payment methods | Simple regulatory and simple financing intervention | Financing and leadership & governance | 20-30% | Reduce |
| Nedberg 2022 | Interrupted time series study | Georgia | Upper middle income country | National level | 2011 to 2021 | 100,000 to 1,000,000 | Not reported | CS rates ceiling and penalty for hospitals exceeding reasonable CS rates | Formulating policy and strategic plans, putting in place levers or tools for implementing policy | Complex regulatory intervention | Leadership & governance, service delivery, health workforce, health information system, financing | 40-50% | Reduce |
| Rosenstein 2021 | Difference in Differences (DID) analysis | United States of America | High income country | Regional/state/provincial level | 2011 to 2021 | more than 1,000,000 | more than 100 | CS rates ceiling, web-based performance matrix, honor rolls for hospital, and incentive for facilities with reasonable CS | Formulating policy and strategic plans, ensuring accountability, putting in place levers or tools for implementing policy, generating intelligence, improvement of quality of care | Complex regulatory intervention | Financing and leadership & governance | 20-30% | Reduce |
| Park 2022 | Interrupted time series study | Republic of Korea (South Korea) | High income country | National level | 2011 to 2021 | 100,000 to 1,000,000 | 50 to 100 | Incentive for facilities for reasonable CS, and penality for providers exceeding reasonable CS rates | Formulating policy and strategic plans, payment methods | Simple regulatory and simple financing intervention | Financing and leadership & governance | Not clear | Increase |
| Liu 2007 | Retrospective before and after study | China (Taiwan) | High income country | National level | Before 2000 | 1000 to 10,000 | Not reported | CS rates ceiling and free births | Formulating policy and strategic plans, benefit design | Simple regulatory and simple financing intervention | Leadership & governance, service delivery, health workforce, health information system | 20-30% | Increase |
| Borem (2020) | Longitudinal study | Brazil | Upper middle income country | Regional/state/provincial level | 2011 to 2021 | 10,000 to 100,000 | 10 to 50 | Multifaceted interventions targeting women and providers involving stakeholder coalition building, involvement of women in decision making, implementation of new care models and improved information systems for continuous learning by health care providers | Formulating policy and strategic plans, improvement of quality of care, ensuring accountability, generating intelligence | Complex regulatory intervention | Financing | More than 50% | Reduce |
| Karami Matin 2018 | Interrupted time series study | Iran (Islamic Republic of) | Lower middle income country | National level | 2011 to 2021 | 10,000 to 100,000 | 10 to 50 | Multifaceted interventions involving CS rates ceiling, incentive for providers with reasonable CS, and free births | Formulating policy and strategic plans, putting in place levers or tools for implementing policy, improvement of quality of care, benefit design | Complex regulatory and simple financial intervention | Financing, leadership & governance, health workforce | 40-50% | Increase |
| Behzadifar 2020 | Interrupted time series study | Iran (Islamic Republic of) | Upper middle income country | National level | 2011 to 2021 | Not reported | 5 to 10 | Multifaceted interventions involving CS rates ceiling, incentive for providers with reasonable CS, and free births | Formulating policy and strategic plans, putting in place levers or tools for implementing policy, improvement of quality of care, benefit design | Complex regulatory and simple financial intervention | Financing, leadership & governance, health workforce | Not clear | Reduce |
| Lotfi 2021 | Interrupted time series study | Iran (Islamic Republic of) | Upper middle income country | National level | 2011 to 2021 | 1000 to 10,000 | Not reported | Multifaceted interventions involving CS rates ceiling, incentive for providers with reasonable CS, and free births | Formulating policy and strategic plans, putting in place levers or tools for implementing policy, improvement of quality of care, benefit design | Complex regulatory and simple financial intervention | Financing, leadership & governance, health workforce | More than 50% | Reduce |
| Pilvar 2021 | Retrospective before and after study | Iran (Islamic Republic of) | Upper middle income country | National level | 2011 to 2021 | Not reported | Not reported | Multifaceted interventions involving CS rates ceiling, incentive for providers with reasonable CS, and free births | Formulating policy and strategic plans, putting in place levers or tools for implementing policy, improvement of quality of care, benefit design | Complex regulatory and simple financial intervention | Financing, leadership & governance, health workforce | 40-50% | Reduce |
| Mosaddeq 2020 | Interrupted time series study | Iran (Islamic Republic of) | Upper middle income country | National level | 2011 to 2021 | more than 1,000,000 | 50 to 100 | Multifaceted interventions involving CS rates ceiling, incentive for providers with reasonable CS, and free births | Formulating policy and strategic plans, putting in place levers or tools for implementing policy, improvement of quality of care, benefit design | Complex regulatory and simple financial intervention | Financing, leadership & governance, health workforce | Not clear | Reduce |
| Parwanehsadeghi 2018 | Retrospective before and after study | Iran (Islamic Republic of) | Upper middle income country | National level | 2011 to 2021 | 1000 to 10,000 | less than 5 | Multifaceted interventions involving CS rates ceiling, incentive for providers with reasonable CS, and free births | \| Formulating policy and strategic plans, putting in place levers or tools for implementing policy, improvement of quality of care, benefit design \| \| --- \| | Complex regulatory and simple financial intervention | Financing, leadership & governance, health workforce | More than 50% | Reduce |
| Rashidian 2019 | Interrupted time series study | Iran (Islamic Republic of) | Upper middle income country | National level | 2011 to 2021 | 100,000 to 1,000,000 | 10 to 50 | Multifaceted interventions involving CS rates ceiling, incentive for providers with reasonable CS, and free births | Formulating policy and strategic plans, putting in place levers or tools for implementing policy, improvement of quality of care, benefit design | Complex regulatory and simple financial intervention | Financing, leadership & governance, health workforce | Not clear | No change |
