# Supplementary material for: Financial and regulatory interventions to reduce unnecessary caesarean sections: An updated scoping review
Source: PLOS Glob Public Health. 2026 Feb 9;6(2):e0005830. doi: 10.1371/journal.pgph.0005830 (PMC12885279; doi:10.1371/journal.pgph.0005830)
Supplement: Attachment — Submitted filename: PGPH-D-25-02141_reviewed document.pdf [file pgph.0005830.s009.pdf]

PLOS Global Public Health

Financial and regulatory interventions to reduce unnecessary caesarean sections: an updated scoping review

--Manuscript Draft--

Manuscript Number:	PGPH-D-25-02141
Article Type:	Research Article
Full Title:	Financial and regulatory interventions to reduce unnecessary caesarean sections: an updated scoping review
Short Title:	Policy interventions to reduce unnecessary caesarean sections: an updated scoping review
Corresponding Author:	Rana Islamiah Zahroh, PhD<br>The University of Melbourne School of Population and Global Health<br>Parkville, Victoria AUSTRALIA
Order of Authors:	Rana Islamiah Zahroh, PhD
	Alya Hazfiarini, MPH
	Martha Vazquez Corona, MPH
	Thiago Melo Santos, PhD
	Nicole Minckas, PhD
	Newton Opiyo, PhD
	Fahdi Dkhimi, MPhil, MPH
	Veloshnee Govender, PhD
	Meghan A. Bohren, PhD
	Ana Pilar Betran, PhD
Keywords:	caesarean section; health system; intervention; financial; regulatory; systematic review
Abstract:	Introduction<br><br>Caesarean section (CS) is a life-saving procedure and critical component of comprehensive obstetric care. However, global CS rates are rising beyond levels justified by clinical indications. Growing evidence suggests health system supply-side factors, such as provider payment models that financially reward CS over vaginal birth and the absence of strict clinical guidelines, are contributing to this trend. Despite growing concerns, there is limited research assessing the implementation and impact of financial and/or regulatory interventions aimed at reducing unnecessary CS.<br><br>Methods<br><br>We conducted this scoping review to update, expand, and synthesise new evidence published since a 2020 scoping review on financial and/or regulatory interventions. We searched MEDLINE, EMBASE, CINAHL, Global Index Medicus, Ebsco MultiDisciplinary Databases to identify eligible studies between 1 January 2019 and 3 September 2024. We searched for sibling studies of included interventions to provide comprehensive information.<br><br>Results<br><br>We included 46 full-text papers, comprising 31 intervention studies and 15 sibling studies, from 24 unique interventions. The number of studies assessing financial and/or regulatory interventions has doubled since the 2020 review. Most interventions were implemented in high-income countries, in settings where baseline CS rates were over 20% at either the regional or national level. Nearly half of the interventions were financial and complex, meaning they integrated multiple context-specific components

Powered by Editorial Manager® and ProduXion Manager® from Aries Systems Corporation

	to address interconnected issues, and primarily targeted hospitals or health workers. Complex regulatory interventions combining mandated guidelines, CS rate thresholds, and penalties or incentives revealed more benefits for birth outcomes than financing or simple regulatory interventions, though they may be costly. Overall, there are still limited studies that combine both financial and regulatory interventions, and health outcomes were typically not assessed in complex financial interventions.<br><br>Conclusion<br><br>Future research should consider context-specific complex interventions and ensure that evaluations include maternal and baby health outcomes, to prevent avoidable harms.
**Additional Information:**	
**Question**	**Response**
**Financial Disclosure**<br><br>Enter a financial disclosure statement that describes the sources of funding for the work included in this submission and the role the funder(s) played. This includes grants and any commercial funding of the work or authors.<br><br>This statement will be typeset if the manuscript is accepted for publication.<br><br>*Please review the submission guidelines and the instructions link below for detailed requirements and guidance.*	This work received funding from the UNDP-UNFPA-UNICEF-WHO-World Bank Special Programme of Research, Development and Research Training in Human Reproduction (HRP), a cosponsored programme executed by the World Health Organization. MAB's time is supported by an Australian National Health and Medical Research Council (NHMRC) Investigator grant (2025634). The views expressed in this article are those of the authors and do not necessarily reflect the funders.
**Competing Interests**<br><br>On behalf of all authors, disclose any competing interests that could be perceived to bias this work.<br><br>This statement will be typeset if the manuscript is accepted for publication.<br><br>*Please review the instructions link below and PLOS Global Public Health's competing interests policy to determine what information must be disclosed at submission.*	No competing interests.
**Data Availability**	This study is based on published articles, all of which are either publicly available or

Before publication, Authors are required to make fully available and without restriction all data underlying their findings. Please see our PLOS Data Policy page for detailed information on this policy.<br><br>A **Data Availability Statement**, detailing where the data can be accessed, is required at first submission. Insert your Data Availability Statement in the box below.<br><br>Please see the data reporting section of our submission guidelines for instructions on what you need to include in your Data Availability Statement.<br><br>This statement will be typeset if the manuscript is accepted for publication.<br><br>*PLOS allows rare exemptions to address legal and ethical concerns. If you have legal or ethical restrictions, please use the box below to detail these in full sentences for the Journal team to consider.*	accessible through academic institutional credentials. The authors confirm that all other relevant data are included within the main text and/or supplementary files.

Manuscript
Click here to access/download;Manuscript;Main document_28072025.docx

Financial and regulatory interventions to reduce unnecessary caesarean sections: an updated scoping review

Authors:

Rana Islamiah Zahroh, PhD[1*]

Alya Hazfiarini, MPH[1]

Martha Vazquez Corona, MPH[1]

Thiago Melo Santos, PhD[1]

Nicole Minckas, PhD[1]

Newton Opiyo, PhD[2]

Fahdi Dkhimi, MPhil, MPH[2]

Veloshnee Govender, PhD[2]

Meghan A. Bohren, PhD[1]

Ana Pilar Betran, PhD[2]

Affiliations:

[1]Gender and Women's Health Unit, Nossal Institute for Global Health, Melbourne School of Population and Global Health, The University of Melbourne, Carlton, Victoria, Australia

[2]UNDP/UNFPA/UNICEF/WHO/World Bank Special Programme of Research, Development and Research Training in Human Reproduction (HRP), Department of Sexual and Reproductive Health and Research, World Health Organization, Geneva, Switzerland.

*Corresponding author: Rana Islamiah Zahroh, r.zahroh@unimelb.edu.au

ABSTRACT

Introduction: Caesarean section (CS) is a life-saving procedure and critical component of comprehensive obstetric care. However, global CS rates are rising beyond levels justified by clinical indications. Growing evidence suggests health system supply-side factors, such as provider payment models that financially reward CS over vaginal birth and the absence of strict clinical guidelines, are contributing to this trend. Despite growing concerns, there is limited research assessing the implementation and impact of financial and/or regulatory interventions aimed at reducing unnecessary CS.

Methods: We conducted this scoping review to update, expand, and synthesise new evidence published since a 2020 scoping review on financial and/or regulatory interventions. We searched MEDLINE, EMBASE, CINAHL, Global Index Medicus, Ebsco MultiDisciplinary Databases to identify eligible studies between 1 January 2019 and 3 September 2024. We searched for sibling studies of included interventions to provide comprehensive information.

Results: We included 46 full-text papers, comprising 31 intervention studies and 15 sibling studies, from 24 unique interventions. The number of studies assessing financial and/or regulatory interventions has doubled since the 2020 review. Most interventions were implemented in high-income countries, in settings where baseline CS rates were over 20% at either the regional or national level. Nearly half of the interventions were financial and complex, meaning they integrated multiple context-specific components to address interconnected issues, and primarily targeted hospitals or health workers. Complex regulatory interventions combining mandated guidelines, CS rate thresholds, and penalties or incentives revealed more benefits for birth outcomes than financing or simple regulatory interventions, though they may be costly. Overall, there are still limited studies that combine both financial and regulatory interventions, and health outcomes were typically not assessed in complex financial interventions.

Conclusion: Future research should consider context-specific complex interventions and ensure that evaluations include maternal and baby health outcomes, to prevent avoidable harms.

Keywords: caesarean section, health system, intervention, financial, regulatory, systematic review

INTRODUCTION

Caesarean section (CS) is a critical life-saving surgical procedure and a key component of comprehensive obstetric care. The World Health Organization (WHO) considers the provision of timely, respectful, and high-quality CS to all women in need as a global priority [1,2]. However, global rates of CS are rising beyond levels justified by clinical need [3,4] with increasing use among populations of women who could be considered low-risk for a CS [4,5]. This trend is projected to persist in the coming years [3], raising concerns about the health risks to women and babies, as well as the health system implications, including increased costs and negative impacts on the quality and accessibility of care [6]. Specifically, non-medically indicated CS (referred to as "unnecessary" CS) has been linked to adverse maternal and neonatal outcomes [5,7].

Supply-side health system factors are significant drivers of high CS rates and can often influence women's (through supplier-induced demand) and health workers' (e.g., obstetricians) clinical decisions [8,9]. For example, payment systems that are output-oriented may incentivise hospitals and health workers to perform more CS, especially where CS is reimbursed at higher rates than vaginal birth [12–14]. The lack of robust audit systems and clinical guidelines on CS, combined with health workers' fears of litigation, may encourage health workers to conduct CS as a defensive practice [13,15]. Furthermore, competing demands, such as practising across multiple hospitals, may influence health workers to favour CS as it is more predictable, and easier to schedule and manage than vaginal birth [12–14].

The influence of the health system and health workers on women's choices and recommendations needs consideration given evidence indicating that most women prefer vaginal births and less than 10% of women actively request CS [10,11]. WHO recommends understanding and addressing these health system factors, especially in low- and middle-income countries where the largest increases in CS rates have been observed [3,16]. This is essential to ensure that CS are performed appropriately, while acknowledging and addressing the power imbalances and hierarchical relationships that exist between health facilities, health workers, and women. [17].

Several reviews have explored health system interventions aimed at reducing unnecessary CS. Chapman et al (2019) [18] and Chen et al (2018) [19] evaluated interventions targeting health workers and health organisations, such as audit and feedback, second opinion for CS decision, and peer review to ensure practice

compliance. Boatin et al (2018) [20] assessed audit and feedback interventions based on the Robson classification, while Torloni et al (2023) [21] assessed interventions to increase or re-introduce assisted vaginal birth (forceps or vacuum). Only two systematic reviews by Yu et al (2019) [22]and Opiyo et al (2020) [8] evaluated both financial and regulatory interventions. However, both included studies were published up to 2018 and 2019 respectively, the findings presented inconsistent intervention effects [8,22], and generally low-quality evidence [8]. More rigorous studies and innovative methods for assessing the impact of financial and regulatory interventions were deemed necessary [8]. In the five years since these previous reviews were published, new evidence may now be available to address these research gaps.

We aimed to update Opiyo et al (2020) [8] scoping review on financial and/or regulatory interventions implemented globally to reduce unnecessary CS , by synthesising new evidence published since the 2020 publication. The specific objectives were to: (i) describe the characteristics of identified financial and/or regulatory interventions intended to reduce unnecessary CS, and (ii) explore the effectiveness, safety, and related outcomes of the identified financial and/or regulatory interventions.

MATERIALS AND METHODS

This scoping review adhered to the Preferred Reporting Items for Systematic Reviews and Meta-analysis extension for scoping reviews (PRISMA-Scr; S1 Appendix) [23]. The protocol is registered with the Open Science Framework (https://osf.io/7btgp).

CRITERIA FOR CONSIDERING STUDIES FOR THIS REVIEW

TYPE OF STUDIES

All randomised trials, non-randomised trials, before-after studies, interrupted time-series studies, cohort studies, large-scale intervention case studies, implementation studies, pre-post intervention studies, cross-sectional, and mixed-methods studies were eligible for inclusion. We included studies published in any language. We excluded non-primary papers (e.g., case reports, letters, editorials, commentaries, reviews, study protocols, posters, and conference abstracts).

TYPES OF PARTICIPANTS

We included studies involving groups of low-risk pregnant women, health workers, and health facilities providing maternity care. Low-risk pregnant women were defined as those receiving antenatal and intrapartum care in health facilities, with term, singleton, and cephalic pregnancies, with or without a previous CS, corresponding to Robson groups 1 through 5 [24]. If the Robson classification was unclear or not explicitly defined, studies were included if the population was likely to pertain to low-risk women. We excluded studies that involved exclusively women with specific medical conditions (e.g., diabetes, obesity, or HIV). Health workers included nurses, midwives, obstetricians, and doctors involved in antenatal or intrapartum care. We included studies conducted in health facilities with childbirth care (e.g., hospitals, birthing centres).

In this scoping review, we use the term “women” to refer to individuals with reproductive capacity for pregnancy and childbirth. We acknowledge that not all people who give birth identify as women and recognise the importance of inclusive language. However, we have chosen to retain the term “women” in this context to highlight the gendered systemic discrimination that women face in maternity care. Gendered expectations, medicalisation, and structural inequities disproportionately affect women’s experiences and access to respectful, evidence-based care. While this does not diminish the experiences of gender-diverse pregnant people, the decision to use “women” reflects the historical and ongoing challenges in maternal health policy, practice, and research.

SETTINGS

We included studies conducted in any country, at any health system level (e.g., national, regional, district) and any type of health facilities (e.g., hospitals, birthing centres, public, private).

TYPE OF INTERVENTIONS

We included studies reporting any financial and/or regulatory interventions, including complex interventions that were either multi-component (comprising multiple interrelated elements within a single intervention) or multi-faceted (targeting different levels such as women, health workers, health facilities, or health systems). Interventions were eligible if they incorporated financial and/or regulatory measures and were aimed at reducing unnecessary CS. We excluded studies aiming to increase CS use. Our focus on interventions intended

to reduce unnecessary procedures reflects growing concern over the global oversupply of CS, their associated health risks, and the strain they place on health systems [6,17].

We defined financial and regulatory interventions according to the WHO health system building blocks (S2 Appendix). Financial interventions refer to the implementation of a strategy or reform in healthcare financing, including those designed to influence service delivery and reduce unnecessary CS. This includes changes in the way health providers are selected and contracted, how and how much they are paid for these services, and what reporting is required in exchange for such payments. Regulatory interventions refer to the implementation of policies, regulations, or legislation, including initiatives to strengthen the role of patients or communities in health governance, designed to reduce the unnecessary use of CS.

TYPE OF OUTCOME MEASURES

All outcomes related to the effectiveness or safety (i.e., maternal and neonatal health outcomes) of the intervention reported in the included studies were extracted, mapped, and reported.

SEARCH METHODS FOR IDENTIFICATION OF STUDIES

We searched MEDLINE, EMBASE, CINAHL, Global Index Medicus, Ebsco MultiDisciplinary Databases to identify eligible studies (S3 Appendix: Search strategy). We used the search strategy from Opiyo et al (2020) [8] review for each database, and searched for studies published between 1 January 2019 to 3 September 2024. Search terms included terms related to CS and relevant financial and regulatory interventions. As we were interested in including complex interventions involving financing and/or regulatory measures, we re-screened all full-text papers excluded by Opiyo et al (2020) [8]. The WHO International Clinical Trials Registry and ClinicalTrials.gov were also searched for any ongoing studies.

We searched for sibling studies of the included interventions for additional information on the characteristics of the interventions or additional outcomes that may not have been presented in the papers retrieved by our searches. Sibling studies were those linked to the main intervention paper but published separately, such as formative research, process evaluations, costing, and cost-effectiveness studies. To locate sibling studies, we reviewed reference lists of all included intervention studies, and conducted a forward reference search using the “Cited by” function in PubMed and Google Scholar. One review author screened potential sibling studies,

which a second review author double-checked. Disagreements were discussed and adjudicated by the third review author if needed.

SELECTION OF STUDIES

The titles and abstracts retrieved from various databases were compiled into a single reference database using EndNote (www.endnote.com), where duplicates were removed. Following deduplication, all references were imported into Covidence (www.covidence.org). We duplicated the Covidence project from the Opiyo et al (2020) [8] review, to not re-screen studies already included in their review. Two independent review authors assessed study eligibility based on the title and abstract against predefined inclusion and exclusion criteria. Studies meeting the inclusion criteria underwent full-text retrieval for further assessment. Studies that did not meet the inclusion criteria were excluded. In cases of disagreement during any stage of screening and assessment, consensus was sought through discussion among the research team.

LANGUAGE TRANSLATION

During full-text screening, we found two studies in Portuguese, two studies in Persian, and one study in Mandarin, which were translated to English using an open-source software, Google Translate, to verify the eligibility. If eligible for inclusion, the full text and the extraction were double reviewed by a native speaker.

DATA EXTRACTION

We developed a specific form for data extraction for this review, including information on study objectives, methodology, design, settings, participant characteristics, and description of intervention. The data extraction was tested and reviewed by all authors before use.

ASSESSING THE QUALITY OF INCLUDED STUDIES

The Cochrane Risk of Bias tool (RoB 2) was used to appraise the quality of the included studies. Domains assessed for risk of bias included confounding, participant selection, intervention classification, adherence to intended interventions, missing data, outcome measurement, and selective reporting. After the risk of bias assessment, two reviewers evaluated the confidence or certainty of the body of evidence for each outcome (referred to as “quality of evidence” or “confidence in the estimate”) using the Grading of Recommendations

Assessment, Development and Evaluation (GRADE) framework. According to this method, the certainty of evidence for each outcome is graded as “High”, “Moderate”, “Low”, or “Very low” based on predefined criteria. The results of the appraisal and confidence assessment were only used to inform the interpretation of results, but not to exclude any studies. Any disagreements between the two reviewers were resolved through discussion with a third reviewer. The results of the assessment are presented in S4 Appendix.

DATA ANALYSIS AND SYNTHESIS

To enable comparison across studies, we categorised all extracted data according to intervention settings using the World Bank income level [25], WHO region classification, and health system level (national, regional, and facility). We further classified intervention characteristics using WHO Health System Building Blocks [26], the target of the intervention (women, health workers, health organisations), and the type of intervention (financial and/or regulatory).

We also categorised interventions as either simple or complex based on adapted criteria from two WHO frameworks: 1) the WHO strategic health purchasing framework to classify health financing interventions [27], and 2) the WHO frameworks on health system governance for universal health coverage and primary healthcare operational to classify regulatory interventions [28,29]. These frameworks define different types of financial and regulatory interventions (Table 1).

Interventions were considered complex if they implemented more than one type of intervention within a single program. For example, shifting from fee-for-service to a global budget was a “simple financing intervention” as it involved only a payment method change (Table 1). In contrast, an intervention that combined laws to enforce clinical guidelines, hospital review boards, and mandatory reporting was a “complex regulatory intervention”, as it involved several types of regulatory interventions listed in Table 1: formulating policy and strategic plans, improving quality care, and ensuring accountability.

Table 1. Types of financial and regulatory interventions.

**Types of financial interventions**
**Benefit design**<br>Any strategies that modify the conditions of access attached to publicly funded healthcare benefits. For example, any changes in copayment or care pathway (e.g. Enforcing referral).

Provider selection processes
Any strategies that focus on selecting the type of providers that are eligible to provide the service. For example, any form of provider selection process (limiting access to public providers, or to a narrower set of providers).

Contracting modalities including reporting obligations

Any strategies that focus on the relationship between payers (e.g., government or insurance bodies) and providers. Examples include mechanisms such as audits, performance targets, and reporting requirements, aimed at enhancing accountability and service quality.

Payment methods
Any strategies that implement the way (how much, how, and when) providers are compensated, aimed at influencing their behaviour and the efficiency of service delivery. Payment methods (e.g., capitation, fee-for-service, case-based payment) are designed to balance cost control.

Types of regulatory interventions

Formulating policy and strategic plans
Any strategies defining governance structures for priority-setting, policy, or legislative mandate, to ensure evidence-based decision-making. Examples include setting national health strategies, policies, guidelines, and protocol implementation.

Generating intelligence
Any strategies designed to produce information for decision-making to improve clinical practice, such as review mechanisms or other activities, as far as the endpoint is to produce information to improve care.

Putting in place levers or tools for implementing policy

Any strategies or activities to address policy barriers and bottlenecks, such as the implementation of incentives or penalties, recognition programs or financial rewards/penalties based on governance targets.

Ensuring accountability
Governance structures or strategies to improve accountability by establishing reporting obligations, setting clear roles and responsibilities, monitoring and evaluation. Example includes the establishment of review boards, agencies or coalitions, regular audit and feedback, peer performance scorecards, publicly accessible CS rates dashboards to track progress, information systems, or other activities to improve accountability.

Improvement of the quality of care
Any strategies for implementing a service or program to improve quality of care that is safe, effective, people-centred, timely, efficient, equitable, and integrated. Which may target any of the following:

- *Health system environment: c*apacity building for health workers, such as training or mentoring, **or** improvement of physical infrastructure, such as enhancing spaces to conduct vaginal births
- *Reducing harm:* implementation of new care models, or activities that uphold foundational principle of causing non avoidable harm to people
- *Improvement in clinical care:* implementation of hospital-based clinical pathways, protocols, context-appropriate standards, checklists, standardised clinical forms and clinical decision support tools
- *Patient, family and community engagement and empowerment:* involvement of relevant stakeholders, including women and their families

We also categorised each reported outcome based on the classifications used in another review [30] to determine the level of impact of each intervention based on effect estimates and certainty of evidence (Table 2). The classification for each study can be seen in S5 Appendix.

After the categorisations were completed, the data were systematically summarised in a descriptive format, qualitatively assessing intervention characteristics and reported outcomes. Findings were synthesised to identify common themes, patterns, and variations across studies. When the same intervention was reported in multiple journal articles or reports, these were merged into a single study, with the unique study being the primary unit of review rather than each report. Furthermore, if multiple studies assessed the same intervention, but reported the components differently, we reported all the components together to get a comprehensive view of the intervention.

Table 2. Outcome classifications adapted by [30]**.**

Category	Effect estimate (RR/OR/WMD)*	Certainty of the evidence	Terminology in text
**Possible benefit**	Evidence or effect of benefit with and without confidence interval (if with, the 95% confidence interval should not cross the line of no effect)	Low	"May reduce/increase… "
**Possible no difference of effect**	Evidence or effect near the line of no effect with and without confidence interval (if with, a narrow 95% confidence interval crossing the line of no effect between 1)	Low	"May have no effect …"
**Possible Harm**	Evidence or effect of harm with and without confidence interval (if with, the 95% confidence interval not crossing the line of no effect)	Low	"May reduce/increase …"
**No conclusion possible**	Any evidence or effect estimates with a wide 95% confidence interval crossing the line of no effect substantially	Low	"There is insufficient evidence …"
	Any evidence or effect estimates	Very low	"It is uncertain whether …"
**No data**	No studies or no data	Not applicable	"No studies …"

*RR: risk ratio, OR: odds ratio, WMD: weighted mean difference.

RESULTS

OVERVIEW OF INCLUDED STUDIES

We identified 4,272 titles and abstracts from database search updates and trial registry searches. After eligibility screening, we included a total of 46 full texts from 31 intervention and 15 sibling studies, reporting on 24 unique interventions (Figure 1). No ongoing clinical trials were found.

Fig 1. PRISMA flowchart.

[insert fig 1]

All studies aimed to evaluate the impact of policies on CS rate reduction (n=31/31, 100%). Forty-five per cent commenced between 2011 and 2021. The most common study designs were retrospective before and after studies (n=14/31;45.2%) and interrupted time series (n=9/31;29.0%). The included studies were mostly conducted in high-income countries (n=20/31;64.5%), with most studies conducted in the United States of America (USA) (n=8/31;25.8%), Iran (n=7/31;22.6%), and Taiwan (n=5/31;16.1%). More than half of the studies analysed >100,000 births (n=18/31;58.1%). Almost half of the studies analysed data in less than 5 (n=6/31;19.4%) or between 10 to 50 (n=4/31;12.9%) health facilities. The most common type of health facility was either a mix of private and public (n=14/31;45.2%) or public (n=7/31;22.6%). An overview of the included studies is shown in Figure 2, and detailed characteristics can be found in S6 Appendix.

Fig 2. Overview of 31 included intervention studies.

[insert fig 2]

Figure 3 shows an overview of the 24 included interventions. The types of interventions implemented were either complex regulatory (n=6/24;25.0%), complex financing (n=6/24;25.0%), or simple financing interventions (20.8%;5/24). Most interventions targeted the health financing building blocks (n=11/24;45.8%). Almost 80% (n=19/24) of the interventions were implemented in settings where baseline CS rates were between 20% and 78%. Most interventions were implemented at either the regional (n=12/24;50.0%) or national levels (n=10/24;41.7%), and more than half targeted either health organisations (n=7/24;29.2%) or health workers (n=6/24;25.0%). Only two interventions adopted theoretical, or behaviour change frameworks during intervention design. Prior to applying the risk of bias and certainty of evidence criteria (Table 2), 54% (n=13/24) of interventions reported a CS reduction, while the remaining showed either an increase (n=7/24;29.2%), no change (n=2/24;8.3%), or mixed effects (n=2/24;8.3%). Most studies had a “not serious”

risk of bias (n=20/31;62.5%), while 28.1% were rated as “serious” (n=8/31) and 9.4% as “very serious” (n=3/24). Due to the heterogeneity across studies and the inability to pool the data, the certainty of evidence was either “low” (n=22/31;71.9%) or “very low” (n=9/31;28.1%) for most studies. See S4 Appendix for more details.

Fig 3. Overview of 24 unique interventions in included studies.

[insert fig 3]

FINANCIAL INTERVENTIONS

SIMPLE FINANCIAL INTERVENTIONS

Five studies evaluated simple financial interventions. Two studies from the USA and China evaluated the change of payment method for health workers and organisations from fee-for-service to blended payment [31] and episode-bundle payment [32] systems. Both studies demonstrated a reduction in CS rates (both low certainty). The remaining three studies equalised the CS and vaginal birth payment rates [33–35]. Two studies from Italy and Taiwan increased the vaginal birth payment rate to match the CS (one-tail equalisation) [33,35], while one study from the USA increased the vaginal birth payment rate while decreasing the CS payment rate to the same level (two-tailed equalization) [34]. Only one study demonstrated a reduction in CS; low certainty [33], while two others did not report clear conclusions; both had very low certainty [34,35]. The successful intervention [33] involved fee equalisation within the Diagnosis-Related Group (DRG) payment system, a model that pays hospitals a fixed amount for patient care based on diagnosis instead of individual services provided. The DRG system had already been implemented before the fee equalisation [33].

COMPLEX FINANCIAL INTERVENTIONS

Seven studies reported complex financial interventions. Two studies evaluated the transition from a fee-for-service to a global budget payment system in Taiwan, with some variations in their approaches [36,37]. Chen et al (2014) [36] analysed the impact of national-level changes, including efforts to increase vaginal birth rate to match CS rate, and also introduction of co-payment by women for non-medically indicated CS. Liu (2013)

[37] analysed the impact of facility-level interventions, along with the implementation of post-CS peer reviews, and audit and feedback. Neither study observed a reduction in CS section, and both were of low certainty.

Two studies evaluated one intervention in Korea, where the DRG payment system was implemented along with changes from a retrospective to a prospective payment system [38,39]. Kim et al (2016) [38] reported a reduction in CS rates (low certainty), while Lee et al (2007) [39] concluded that no change was observed (low certainty). Furthermore, one study implemented a capitation-based system along with removing co-payments for women in China [40], and another with limiting provider selection in the USA [41]. Both increased CS (very low certainty for Chen et al (2016) [40] and low certainty for Misra (2008) [41]. Lastly, Tsai et al (2006) [42] evaluated the implementation of a case-based payment system in Taiwan along with performance incentives for health facilities. The study reported a reduction in CS, but findings were inconclusive due to very low certainty.

REGULATORY INTERVENTIONS

SIMPLE REGULATORY INTERVENTIONS

Three simple regulatory intervention studies from the USA implemented different policies. Cozzi-Glaser et al (2024) [43] evaluated an institutional policy universally offering elective induction of labour at 39 weeks of gestation. Snowden et al (2020) [44] evaluated a state-wide "Hard Stop" policy limiting elective induction of labour and CS before 39 weeks of gestation. Safrin (2023) [45] evaluated the implementation of state-wide damage caps to limit health workers' liability in the case of litigation. Policies related to elective induction of labour reported possible CS reduction, but both low certainty [43,46], and no conclusions could be drawn about the impact of damage caps due to very low certainty [45].

COMPLEX REGULATORY INTERVENTIONS

Six studies evaluated the impact of complex regulatory interventions. Yu et al (2017) [47] evaluated state policies that required health workers to encourage women to choose vaginal birth in Wenzhou, China, which included controlling indications for CS through the implementation of CS rates threshold, and special consent for maternal request for CS. Alongside, health facilities implemented training, guidelines, audit and feedback, penalties for health workers exceeding reasonable CS rates, and health education for women [47]. Nedberg et

al (2022) [48] evaluated a national policy in Georgia aimed at reducing CS by imposing financial penalties for hospitals that did not meet their reduction targets. Rosenstein et al (2021) [49] assessed a multi-faceted quality of care improvement collaboration in the USA that established CS rate thresholds, audit and feedback, public reporting, web-based performance metrics, women's engagement, and service improvements. The collaboration also provided incentives, including an annual honour roll for health workers and fee waivers for hospitals. All three initiatives resulted in reduced CS rates, with low certainty [47–49].

Escuriet-Peiro et al (2015) [50] evaluated the Strategy Paper on Normal Childbirth in Catalonia, Spain, to promote vaginal birth, including financial aid for infrastructure improvement, health workers' training, protocol for vaginal birth, and women's involvement in decision-making. Studnicki et al (1997) [51] evaluated the implementation of practice guidelines in the USA, mandated by legislation, and complemented by hospital peer-review boards and state reporting requirements. In Brazil, Borem et al (2020) [52] introduced quality improvement initiatives that included policies to respect women's birth plans, training health workers, involving women in decision-making, implementing new care models, supporting birth companions, and providing prenatal information on birth options, alongside stakeholder coalition and real-time CS rate monitoring systems. Borem et al (2020) [52] and Studnicki et al (1997) [51] demonstrated success in reducing CS rates (both low certainty), while Escuriet-Peiro (2015) [50] demonstrated an increase in CS (very low certainty).

COMBINATION OF FINANCIAL AND REGULATORY INTERVENTIONS

We only identified two combinations between financial and regulatory interventions, which are: (1) simple regulatory and simple financing interventions, and (2) complex regulatory and simple financial interventions. We did not find any interventions that combine complex financing with simple regulatory interventions.

SIMPLE REGULATORY AND SIMPLE FINANCING INTERVENTIONS

Three studies combined simple regulatory and simple financing interventions. These interventions were categorised differently from complex regulatory interventions, as the financing component directly influenced health workers and/or the hospital's payment. Sukru Budak et al (2020) [53] evaluated a Turkish Ministry of Health-issued policy linking health workers' compensation to their primary CS rate, with full payment when

health workers' CS rates were 15% or less and reduced payment for higher rates. Park et al (2022) [54] evaluated the impact of the Korea Value Incentive Program, which provided incentives and penalties via a 1-5% fee increase for high-performing hospitals and a 1-5% fee reduction for underperforming hospitals). Liu et al (2007) [55] evaluated Taiwan's policy that capped CS rates at 30%, along with hospital accreditation guidelines, and introduced a nearly zero-cost childbirth option for women. Park et al (2022) [54] and Liu et al (2007) [55] resulted in increased CS rates and were both of low certainty. Sukru Budak et al (2020) [53] demonstrated a reduced CS rate, albeit with very low certainty.

COMPLEX REGULATORY AND SIMPLE FINANCIAL INTERVENTIONS

Seven studies evaluated and reported on the effects of implementing one complex regulatory and simple financial intervention: the Iranian Health Transformation Plan, which set a CS rate threshold, provided incentives for health workers through bonus payments, removed co-payment by women, and improved maternity infrastructure and services. However, the outcomes varied across the seven studies, which were conducted in over 100 different health facilities. Two studies showed reduced CS rates; both with low certainty [56,57], one showed increased CS rates; with low certainty [58], one found no change in CS rates; with low certainty [59], and three did not yield conclusive results due to very low certainty [60–62].

INTERVENTION OUTCOMES

Outcomes reported included overall CS rates (n=24/24;100%), rates of different types of CS or vaginal birth (pre-labour CS, induced labour, vaginal birth after CS, or repeat CS; n=13/24, 54.2%), and maternal or neonatal morbidities and mortality (n=12/24;50%). Only five studies reported cost-related outcomes (n=5/24;20.8%), one study (n=1/24;4.2%) reported assisted vaginal birth outcomes, one study (n=1/24;4.2%) reported CS rates by Robson classification, and one study (n=1/24;4.2%) reported women's experiences of and perceptions about CS (Figure 4).

Figure 4 shows the outcomes reported by the studies across different groups of interventions. Overall, studies evaluating complex regulatory interventions assessed more outcomes and revealed more beneficial results regarding mode of birth and maternal and neonatal health, compared to other types of interventions. Complex regulatory interventions demonstrated reductions in the overall CS rate (5/6 studies), repeat CS (1/6 study),

elective CS (1/6 study), increase in induced labour (2/6 studies), vaginal birth (1/6 study), and assisted vaginal birth rates (1/6 study). Complex regulatory interventions are also observed to have benefits on maternal and neonatal outcomes, including reduced maternal mortality (1/6 study), maternal satisfaction (1/6 study), infection rates (1/6 study), neonatal mortality (1/6 study) and an increase in babies born at term and with higher birth weights (1/6 study). Potential harms from increased neonatal respiratory complications were reported in one study. Only one study on complex regulatory interventions reported the cost of birth and cost-effectiveness outcomes, concluding that the intervention was expensive and not cost-effective. Successful complex regulatory interventions implemented policies or guidelines to reduce unnecessary CS, established CS rate thresholds, and enforced penalties or incentives to ensure hospitals and health workers met their targets [47–49,51].

Fig 4. Outcomes across groups of interventions.

[insert fig 4]

Simple financing interventions appeared promising for overall CS rate reduction (3/5 studies). One study also revealed possible benefits in reducing hospitalisations; another study, however, revealed a possible increase in the risk of postpartum haemorrhage. The cost-related outcomes were contradictory, with one study demonstrating benefits and one study showing harm. Successful simple financing interventions often involved reforming the payment system from fee-for-service to specific payment systems. This included blended payments [31], episode-bundle payments [32], and equalisation of the mode of birth fees in the context of DRG payment systems [33].

Simple regulatory interventions demonstrated reductions in overall (2/3 studies) and elective CS (1/3 study) yet appear to be associated with either no significant impact or, in some cases, potential harm to maternal and neonatal outcomes (Figure 4), such as postpartum haemorrhage (2/3 studies), peripartum infection (2/3 studies), and neonatal respiratory complications (1/3 studies). The two successful, simple regulatory interventions, which appear to increase harm (based on low-quality evidence), are related to induction of labour policies [43,46]. Overall, there are limited studies that combine financial and regulatory interventions aimed at reducing unnecessary CS, and safety outcomes were not evaluated in complex financial interventions.

DISCUSSION

The number of studies evaluating financial and/or regulatory interventions has doubled since the publication of the Opiyo et al (2020) [12] review, indicating growing interest and search of innovation in the field. We found that most studies used retrospective before-and-after designs, evaluated policies on financial reform, and were often complex interventions. Most interventions were implemented in high-income countries, in settings where baseline CS rates were over 20% at either the regional or national level. Most targeted either health organisations or health workers. Complex regulatory interventions that mandated policies or non-clinical guidelines to reduce unnecessary CS, established CS rate thresholds, and enforced penalties or incentives to ensure hospitals and health workers met their targets appear most promising to reduce CS rates and ensure safety. However, these interventions were costly. Simple financial interventions demonstrated the impact of CS rate reductions, but lacked data on safety. Few studies combined financial and regulatory interventions to reduce unnecessary CS, and safety outcomes were not assessed in complex financial interventions.

Reducing unnecessary CS requires multi-component and multi-faceted interventions because the factors driving high rates are complex and operate at multiple levels, including individual, facility, and health system levels [17]. Single-component interventions may fail to achieve or sustain reductions because they do not address the broader organisational, cultural, and contextual factors that influence clinical decision-making. A previous study on interventions targeting health workers has also reported that five important interventions need to be implemented in a single intervention/program together to promote success of the interventions in reducing CS [63]. This is aligned with the WHO statement that “multi-faceted (rather than single-component) interventions tailored to local determinants (barriers and facilitators) of CS practices are recommended” to reduce unnecessary CS [64]. In this scoping review, studies with complex regulatory interventions assessed more outcomes and showed greater benefits in mode of birth (e.g., reduced CS rates, increased vaginal birth) and maternal and neonatal health (e.g., reduced mortality, infection, haemorrhage), compared to simple financial, complex financial, and simple regulatory interventions.

Key complex regulatory interventions to reduce unnecessary CS identified in this review include the implementation of mandated policies or non-clinical guidelines, established CS rate thresholds, and enforced

penalties or incentives to ensure hospitals and health workers met their targets. This further supports previous studies that have emphasised the need for mandated policies or guidelines to change health workers' practice, as they provide a clear and evidence-based framework, improved knowledge and professional competency, which then improves decision-making about CS [63,65]. The practice of establishing CS rate thresholds and imposing penalties or incentives for health workers who are over or under the threshold may create strong accountability mechanisms that encourage adherence to guidelines and motivate health workers to make evidence-based decisions [28,29]. However, these practices could also result in unintended consequences, such as health workers opting for vaginal births when a CS is clinically indicated, compromising the quality of care and safety of women and babies [48].

Our review highlights the importance of considering potential unintended effects on women's and babies' safety when implementing interventions to reduce CS rates. Several included studies, assessing simple regulatory and simple financial interventions, reported increases in adverse maternal outcomes (postpartum haemorrhage, maternal blood transfusion, chorioamnionitis) when CS was reduced [31,43,46]. These adverse outcomes may be attributable to longer or more complex labours resulting from shifts away from CS, particularly in cases of induction of labour or efforts to support vaginal birth in borderline or high-risk scenarios [31,43,46]. This suggests that some interventions may lack the specificity or sensitivity needed to distinguish between necessary and unnecessary CS. In some settings, increased awareness and changes in clinical protocols may also have contributed to higher reporting rates of these outcomes [31,43,44,46]. These findings highlight that a blunt reduction in overall CS rates, while potentially appealing as a policy target, risks excluding women who genuinely need the procedure. Such interventions may inadvertently reduce access for the most vulnerable, rather than curbing truly unnecessary CS. A safe reduction of CS rates should be accompanied by enhanced labour management and monitoring systems for continuous evaluation and reporting, to ensure that improvements in one area do not lead to harm in another and do not compromise timely access to women who require the procedure for medical reasons [2]. Therefore, the impact of interventions on risks and safety should be continuously monitored and evaluated during implementation. It is crucial to ensure that efforts to reduce unnecessary CS do not compromise timely access for women who require the procedure for medical reasons [2].

Our review shows that system-based financial reform from fee-for-service to certain payment systems (DRG, global budget payment, case-based reimbursement, etc) seems to have a greater impact in reducing CS rates compared to equalising vaginal birth and CS fees. This is aligned with findings from a related review [22], which found that simple economic incentive interventions were less effective in influencing health workers and may suggest that financial interventions must be substantial, system-oriented, and dynamic to drive meaningful change. Our review suggests that it is unlikely for a single purchasing instrument or payment mechanism to be a silver bullet. To influence health workers' behaviour effectively, purchasing arrangements, including payment methods, payment rates, and contracting modalities, need to be continuously adapted. The experience from Taiwan offers a powerful example of this. Initially, Taiwan equalised payment rates by aligning the fees for vaginal births with those for CS [35,42,55], which was effective in reducing CS rates. However, a few years later, the system faced growing concerns around cost containment. This led to the introduction of a new set of measures primarily aimed at controlling expenditure [36,37]. This timeline highlights how an intervention that achieves one policy objective can inadvertently create new challenges, underscoring the need for continuous monitoring and thoughtful course correction. However, the scale and timing of reforms matter. While policies must be responsive to emerging issues, national-level reforms often require longer timeframes to yield results, and overly frequent adjustments can create confusion or weaken implementation. Policymakers should aim for a balance between adaptability and stability when designing and evaluating reform efforts.

Implications for practice and rese arch

To strengthen the evidence for reducing unnecessary CS, future research should prioritise high-quality studies evaluating the effectiveness of financial and regulatory interventions. More rigorous study designs are needed to establish causality and measure long-term outcomes. Researchers should provide relevant information on background and settings (e.g., overall CS rates, rural versus urban location, birth rates, CS rates), and report detailed health and safety outcomes. The outcomes should not only include the impact on the mode of birth but also the perspectives of women and health workers, and cost-effectiveness. Lastly, more research is needed to provide evidence of such interventions in middle- and low-income countries. Figure 4 shows that financial interventions did not frequently report safety outcomes, compared to regulatory interventions, which are critical to ensure that the CS rate reductions do not increase harm to women and babies. Therefore, financing intervention studies should assess the impact on maternal and neonatal health outcomes (including

mode of birth), as well as quality and safeguards to prevent unintended risks such as delayed medically necessary CS.

Furthermore, very few studies involved women and health workers in the design and evaluation of the interventions. Socioeconomic and cultural factors within society and the health system play a significant role in high CS rates; thus, multidisciplinary collaborations across stakeholder groups are crucial for the reduction of CS [63,66]. Effective interventions require engagement from health workers, policymakers, women, and community organisations. Implementers must also maintain flexibility, adapting programs based on feedback from women and health workers to ensure adoption and adherence [15,67,68]. The involvement of health workers and women in designing and implementing these interventions fosters buy-in and long-term adherence [15,67,68]. Lastly, a few interventions used theoretical or behaviour change frameworks. These frameworks can provide a structured approach to understanding the complex factors influencing CS rates and designing theory-informed interventions that can enhance feasibility, acceptability, and effectiveness [69,70]. Future research should prioritise the integration of such frameworks to strengthen the design, implementation, and impact of interventions aimed at optimising CS use.

Strengths and limitations of the study

This review provides new and up-to-date advancements in financial and/or regulatory interventions to reduce CS. We conducted a sibling studies search to obtain comprehensive information about the interventions. WHO frameworks were used to ensure rigour in defining and categorising the interventions. We visualised the outcomes using a bubble chart to compare which interventions have the most impact. Despite these strengths, this review has some limitations. The studies were varied in terms of designs and type of outcomes, and generally had low quality. Thus, it was not possible to conduct a pooled analysis, and all were assessed as low or very low certainty so effectiveness should be interpreted with caution. Given the small number of studies by intervention type, we cannot ascertain which specific interventions are most effective for each intervention type. Furthermore, as health system interventions become more complex and multi-component, it becomes increasingly challenging to isolate effects and rigorously measure impact, raising concerns around measurability bias.

CONCLUSION

Despite the paucity of high-quality studies on financial and regulatory interventions to reduce unnecessary CS, current evidence allows for several important observations. Firstly, complex regulatory interventions with mandated policies or non-clinical guidelines, the establishment of CS rate thresholds, and the enforcement of penalties or incentives appear to be more effective in reducing CS and enhancing safety. However, these interventions may be costly. Secondly, our review suggests that shifting from fee-for-service to alternative payment models (e.g., DRGs, global budgets, case-based payments) is more effective in reducing CS rates than equalising fees between vaginal births and CS. However, evidence on safety remains limited. These financial reforms must be supported by regulatory measures, including regular clinical audits, to ensure the quality and safety of care. The rise in non-clinically indicated caesarean sections is driven by complex and evolving factors on both the demand side (such as the preferences and expectations of women and their families) and the supply side (including provider behaviour and systemic incentives), the latter being the focus of this paper. While evidence shows that financial incentives can significantly influence clinical decision-making, sometimes contributing to supplier-induced demand, these incentives are not static. A payment rate that motivates providers today may become less effective over time if broader health system dynamics are not considered. Addressing both the clinical and systemic consequences, such as increased health risks for women and newborns, rising costs, inefficiencies, and reduced quality and access to care, requires a health system that is agile and responsive to both supply- and demand-side pressures, guided by ongoing evaluation and continuous feedback. Future research should focus on context-specific complex interventions and ensure evaluation not only on the mode of birth but also on health outcomes, to ensure no harms are done to women and babies. Furthermore, employing rigorous study designs that also support patient choice and ensure ethical considerations are prioritised is essential to establish causality and evaluate long-term outcomes. It is also important to understand the theory of change underpinning each intervention, as responses to certain interventions or incentives may vary across populations and health systems.

Acknowledgements: We extend our thanks to Reihaneh Moniri for double reviewing the extraction of Persian studies, and Patrick Condron and Dr Steve McDonald for their support on the search strategy update.

REFERENCES

[1] Betran A, Torloni M, Zhang J, Gülmezoglu A, Section the WWG on C. WHO Statement on Caesarean Section Rates. BJOG: An International Journal of Obstetrics & Gynaecology 2016;123:667–70. https://doi.org/10.1111/1471-0528.13526.

[2] World Health Organization. WHO statement on caesarean section rates. 2015.

[3] Betran AP, Ye J, Moller A-B, Souza JP, Zhang J. Trends and projections of caesarean section rates: global and regional estimates. BMJ Global Health 2021;6:e005671. https://doi.org/10.1136/bmjgh-2021-005671.

[4] Vogel JP, Betrán AP, Vindevoghel N, Souza JP, Torloni MR, Zhang J, et al. Use of the Robson classification to assess caesarean section trends in 21 countries: a secondary analysis of two WHO multicountry surveys. Lancet Glob Health 2015;3:e260-270. https://doi.org/10.1016/S2214-109X(15)70094-X.

[5] Souza J, Gülmezoglu A, Lumbiganon P, Laopaiboon M, Carroli G, Fawole B, et al. Caesarean section without medical indications is associated with an increased risk of adverse short-term maternal outcomes: the 2004-2008 WHO Global Survey on Maternal and Perinatal Health. BMC Med 2010;8:71. https://doi.org/10.1186/1741-7015-8-71.

[6] Sandall J, Tribe RM, Avery L, Mola G, Visser GH, Homer CS, et al. Short-term and long-term effects of caesarean section on the health of women and children. Lancet 2018;392:1349–57. https://doi.org/10.1016/S0140-6736(18)31930-5.

[7] Lavender T, Hofmeyr GJ, Neilson JP, Kingdon C, Gyte GM. Caesarean section for non-medical reasons at term. Cochrane Database Syst Rev 2012;2012:CD004660. https://doi.org/10.1002/14651858.CD004660.pub3.

[8] Opiyo N, Young C, Requejo JH, Erdman J, Bales S, Betrán AP. Reducing unnecessary caesarean sections: scoping review of financial and regulatory interventions. Reproductive Health 2020.

[9] Zahroh RI, Hazfiarini A, Martiningtyas MA, Ekawati FM, Emilia O, Cheong M, et al. Rising caesarean section rates and factors affecting women's decision-making about mode of birth in Indonesia: a longitudinal qualitative study. BMJ Global Health 2024;9:e014602. https://doi.org/10.1136/bmjgh-2023-014602.

[10] Etcheverry C, Betrán AP, de Loenzien M, Robson M, Kaboré C, Lumbiganon P, et al. How does hospital organisation influence the use of caesarean sections in low- and middle-income countries? A cross-sectional survey in Argentina, Burkina Faso, Thailand and Vietnam for the QUALI-DEC project. BMC Pregnancy & Childbirth 2024;24:1–16. https://doi.org/10.1186/s12884-024-06257-w.

[11] Panda S, Begley C, Daly D. Influence of women's request and preference on the rising rate of caesarean section – a comparison of reviews. Midwifery 2020;88:N.PAG-N.PAG. https://doi.org/10.1016/j.midw.2020.102765.

[12] Opiyo N, Kingdon C, Oladapo OT, Souza JP, Vogel JP, Bonet M, et al. Non-clinical interventions to reduce unnecessary caesarean sections: WHO recommendations. Bulletin of the World Health Organization 2020;98:66–8. https://doi.org/10.2471/BLT.19.236729.

[13] Panda S, Begley C, Daly D. Clinicians' views of factors influencing decision-making for caesarean section: A systematic review and metasynthesis of qualitative, quantitative and mixed methods studies. PLoS One 2018;13. https://doi.org/10.1371/journal.pone.0200941.

[14] Kingdon C, Downe S, Betran AP. Non-clinical interventions to reduce unnecessary caesarean section targeted at organisations, facilities and systems: Systematic review of qualitative studies. PLOS ONE 2018;13:e0203274. https://doi.org/10.1371/journal.pone.0203274.

[15] Kingdon C, Downe S, Betran AP. Interventions targeted at health professionals to reduce unnecessary caesarean sections: a qualitative evidence synthesis. BMJ Open 2018;8:e025073. https://doi.org/10.1136/bmjopen-2018-025073.

[16] Bohren MA, Opiyo N, Kingdon C, Downe S, Betrán AP. Optimising the use of caesarean section: a generic formative research protocol for implementation preparation. Reproductive Health 2019;16:170. https://doi.org/10.1186/s12978-019-0827-1.

[17] Betrán AP, Temmerman M, Kingdon C, Mohiddin A, Opiyo N, Torloni MR, et al. Interventions to reduce unnecessary caesarean sections in healthy women and babies. The Lancet 2018;392:1358–68. https://doi.org/10.1016/S0140-6736(18)31927-5.

[18] Chapman A, Nagle C, Bick D, Lindberg R, Kent B, Calache J, et al. Maternity service organisational interventions that aim to reduce caesarean section: a systematic review and meta-analyses. BMC Pregnancy and Childbirth 2019;19:206. https://doi.org/10.1186/s12884-019-2351-2.

[19] Chen I, Opiyo N, Tavender E, Mortazhejri S, Rader T, Petkovic J, et al. Non-clinical interventions for reducing unnecessary caesarean section. Cochrane Database of Systematic Reviews 2018. https://doi.org/10.1002/14651858.CD005528.pub3.

[20] Boatin AA, Cullinane F, Torloni MR, Betrán AP. Audit and feedback using the Robson classification to reduce caesarean section rates: a systematic review. BJOG 2018;125:36–42. https://doi.org/10.1111/1471-0528.14774.

[21] Torloni MR, Opiyo N, Altieri E, Sobhy S, Thangaratinam S, Nolens B, et al. Interventions to reintroduce or increase assisted vaginal births: a systematic review of the literature. BMJ Open 2023;13:e070640. https://doi.org/10.1136/bmjopen-2022-070640.

[22] Yu Y, Lin F, Dong W, Li H, Zhang X, Chen C. The effectiveness of financial intervention strategies for reducing caesarean section rates: a systematic review. BMC Public Health 2019;19:1080. https://doi.org/10.1186/s12889-019-7265-4.

[23] Tricco AC, Lillie E, Zarin W, O'Brien KK, Colquhoun H, Levac D, et al. PRISMA Extension for Scoping Reviews (PRISMA-ScR): Checklist and Explanation. Ann Intern Med 2018;169:467–73. https://doi.org/10.7326/M18-0850.

[24] World Health Organization. Robson Classification: Implementation Manual. World Health Organization; 2017.

[25] World Bank. The World by Income and Region 2023. https://datatopics.worldbank.org/world-development-indicators/the-world-by-income-and-region.html (accessed March 2, 2025).

[26] World Health Organization. Monitoring the Building Blocks of Health Systems: A Handbook of Indicators and Their Measurement Strategies. World Health Organization; 2011.

[27] World Health Organization. Governance for strategic purchasing: An analytical framework to guide a country assessment 2019. https://www.who.int/publications/i/item/9789240000025 (accessed February 9, 2025).

[28] World Health Organization. Health systems governance for universal health coverage action plan: department of health systems governance and financing 2014. https://www.who.int/publications/i/item/WHO-HSS-HSF-2014.01 (accessed February 9, 2025).

[29] World Health Organization. Operational Framework for Primary Health Care 2020. https://www.who.int/publications/i/item/9789240017832 (accessed February 9, 2025).

[30] Gialdini C, Chamillard M, Diaz V, Pasquale J, Thangaratinam S, Abalos E, et al. Evidence-based surgical procedures to optimize caesarean outcomes: an overview of systematic reviews. eClinicalMedicine 2024;72. https://doi.org/10.1016/j.eclinm.2024.102632.

[31] Kozhimannil KB, Graves AJ, Ecklund AM, Shah N, Aggarwal R, Snowden JM. Cesarean Delivery Rates and Costs of Childbirth in a State Medicaid Program After Implementation of a Blended Payment Policy. Med Care 2018;56:658–64. https://doi.org/10.1097/MLR.0000000000000937.

[32] Meng Z, Zou K, Ding N, Zhu M, Cai Y, Wu H. Cesarean delivery rates, costs and readmission of childbirth in the new cooperative medical scheme after implementation of an episode-based bundled payment (EBP) policy. BMC Public Health 2019;19:557. https://doi.org/10.1186/s12889-019-6962-3.

[33] Barili E, Bertoli P, Grembi V. Fee equalization and appropriate health care. Econ Hum Biol 2021;41:100981. https://doi.org/10.1016/j.ehb.2021.100981.

[34] Keeler EB, Fok T. Equalizing physician fees had little effect on cesarean rates. Medical Care Research and Review : MCRR 1996;53:465–71.

[35] Lo JC. Financial incentives do not always work—An example of cesarean sections in Taiwan. Health Policy 2008;88:121–9. https://doi.org/10.1016/j.healthpol.2008.02.013.

[36] Chen C-S, Liu T-C, Chen B, Lin C-L. The failure of financial incentive? The seemingly inexorable rise of cesarean section. Social Science & Medicine 2014:47. https://doi.org/10.1016/j.socscimed.2013.11.010.

[37] Liu C-M, Lin Y-J, Su Y-Y, Chang S-D, Cheng P-J. Impact of health policy based on the self-management program on Cesarean section rate at a tertiary hospital in Taiwan. Journal of the Formosan Medical Association 2013;112:93–8.

[38] Kim SJ, Han K-T, Kim SJ, Park E-C, Park HK. Impact of a diagnosis-related group payment system on cesarean section in Korea. Health Policy 2016;120:596–603. https://doi.org/10.1016/j.healthpol.2016.04.018.

[39] Lee K, Lee S. Effects of the DRG-based prospective payment system operated by the voluntarily participating providers on the cesarean section rates in Korea. Health Policy 2007;81:300–8. https://doi.org/10.1016/j.healthpol.2006.05.019.

[40] Chen C, Cheng Z, Jiang P, Sun M, Zhang Q, Lv J. Effect of the new maternity insurance scheme on medical expenditures for caesarean delivery in Wuxi, China: a retrospective pre/post-reform case study. Front Med 2016;10:473–80. https://doi.org/10.1007/s11684-016-0479-2.

[41] Misra A. Impact of the HealthChoice program on cesarean section and vaginal birth after C-section deliveries: a retrospective analysis. Matern Child Health J 2008;12:266–74. https://doi.org/10.1007/s10995-007-0234-z.

[42] Tsai Y-H, Huang K-C, Soong Y-K. Impact of case payment on physicians practicing vaginal birth after cesarean section. Taiwan Journal of Public Health 2006;25:283–92.

[43] Cozzi-Glaser GD, Blanchard CT, Stanford JN, Oben AG, Jauk VC, Szychowski JM, et al. Outcomes in low-risk patients before and after an institutional policy offering 39-week elective induction of labor. J Matern Fetal Neonatal Med 2024;37:2295223. https://doi.org/10.1080/14767058.2023.2295223.

[44] Snowden JM, Osmundson SS, Kaufman M, Blauer Peterson C, Kozhimannil KB. Cesarean birth and maternal morbidity among Black women and White women after implementation of a blended payment policy. Health Serv Res 2020;55:729–40. https://doi.org/10.1111/1475-6773.13319.

[45] Safrin S. The Impact of Malpractice Damage Caps on C-section Rates and Alternative Approaches. Clin Obstet Gynecol 2023;66:278–92. https://doi.org/10.1097/grf.0000000000000780.

[46] Snowden JM, Muoto I, Darney BG, Quigley B, Tomlinson MW, Neilson D, et al. Oregon's Hard-Stop Policy Limiting Elective Early-Term Deliveries: Association With Obstetric Procedure Use and Health Outcomes. Obstetrics and Gynecology 2016;128:1389–96.

[47] Yu Y, Zhang X, Sun C, Zhou H, Zhang Q, Chen C. Reducing the rate of cesarean delivery on maternal request through institutional and policy interventions in Wenzhou, China. PLoS ONE 2017;12:1–12. https://doi.org/10.1371/journal.pone.0186304.

[48] Nedberg IH, Manjavidze T, Rylander C, Blix E, Skjeldestad FE, Anda EE. Changes in cesarean section rates after introduction of a punitive financial policy in Georgia: A population-based registry study 2017-2019. PLoS One 2022;17:e0271491. https://doi.org/10.1371/journal.pone.0271491.

[49] Rosenstein MG, Chang S-C, Sakowski C, Markow C, Teleki S, Lang L, et al. Hospital Quality Improvement Interventions, Statewide Policy Initiatives, and Rates of Cesarean Delivery for Nulliparous, Term, Singleton, Vertex Births in California. JAMA: Journal of the American Medical Association 2021;325:1631–9. https://doi.org/10.1001/jama.2021.3816.

[50] Escuriet-Peiro R, Goberna-Tricas J, Pueyo-Sanchez MJ, Garriga-Comas N, Ubeda-Bonet I, Caja-Lopez C, et al. Impact of maternity care policy in Catalonia: a retrospective cross-sectional study of service delivery in public and private hospitals. BMC Pregnancy and Childbirth 2015;15:23. https://doi.org/10.1186/s12884-015-0446-y.

[51] Studnicki J, Remmel R, Campbell R, Werner DC. The impact of legislatively imposed practice guidelines on cesarean section rates: the Florida experience. American Journal of Medical Quality : The Official Journal of the American College of Medical Quality 1997;12:62–8.

[52] Borem P, de Cássia Sanchez R, Torres J, Delgado P, Petenate AJ, Peres D, et al. A Quality Improvement Initiative to Increase the Frequency of Vaginal Delivery in Brazilian Hospitals. Obstetrics & Gynecology 2020;135:415–25. https://doi.org/10.1097/AOG.0000000000003619.

[53] Sukru Budak M, Temur M. A national measure to reduce primary cesarean rates in Turkey. J Matern Fetal Neonatal Med 2020;33:1643–6. https://doi.org/10.1080/14767058.2018.1526901.

[54] Park Y, Kim J, Lee K. Changes in cesarean section rate before and after the end of the Korean Value Incentive Program. Medicine 2022;101:e29952–e29952. https://doi.org/10.1097/MD.0000000000029952.

[55] Liu T-C, Chen C-S, Tsai Y-W, Lin H-C. Taiwan’s high rate of cesarean births: impacts of national health insurance and fetal gender preference. Birth (Berkeley, Calif) 2007;34:115–22.

[56] Pilvar H, Yousefi K. Changing physicians’ incentives to control the C-section rate: Evidence from a major health care reform in Iran. J Health Econ 2021;79:102514. https://doi.org/10.1016/j.jhealeco.2021.102514.

[57] Ali Mohammad Mosaddegh Qur’an, Maryam Tajour, Qasem Jan Babaei, Mahbubeh Parsaian, Farah Babaei, Leila Islambolchi. The impact of the Natural Childbirth Promotion Program under the Health System Transformation Plan on Caesarean Section Rates in Iran: A Discontinuous Time Series Study. Hayat / Ḥayāt 2020;26:144–62.

[58] Karami Matin B, Hajizadeh M, Najafi F, Homaie Rad E, Piroozi B, Rezaei S. The impact of health sector evolution plan on hospitalization and cesarean section rates in Iran: an interrupted time series analysis. International Journal for Quality in Health Care : Journal of the International Society for Quality in Health Care 2018;30:75–9. https://doi.org/10.1093/intqhc/mzx169.

[59] Rashidian A, Moradi G, Takian A, Sakha MA, Salavati S, Faraji O, et al. Effects of the Health Transformation Plan on caesarean section rate in the Islamic Republic of Iran: an interrupted time series. East Mediterr Health J 2019;25:254–61. https://doi.org/10.26719/emhj.18.044.

[60] Behzadifar M, Behzadifar M, Saki M, Valipour M, Omidifar R, Iranshahi F, et al. The impact of the “Health Transformation Plan” and related policies on the prevalence rate of cesarean section in Iran: Insights from an interrupted time series analysis. Int J Health Plann Manage 2020;35:339–45. https://doi.org/10.1002/hpm.2916.

[61] Lotfi F, Lohivash S, Kavosi Z, Owjimehr S, Bayati M. The impact of Health Transformation Plan on natural vaginal delivery and cesarean section frequency in Iran: an interrupted time series analysis. BMC Res Notes 2021;14:257. https://doi.org/10.1186/s13104-021-05677-7.

[62] Parvaneh Sadeghi M, Azadeh A, Muneerah T, Hura T, Mohammad AA. The impact of the Health System Transformation Plan on neonatal outcomes: A case study in a teaching hospital, 2012–2016. Qom University of Medical Sciences Journal 2018;12:48–54.

[63] Zahroh RI, Kneale D, Sutcliffe K, Vazquez Corona M, Opiyo N, Homer CSE, et al. Interventions targeting healthcare providers to optimise use of caesarean section: a qualitative comparative analysis to identify

important intervention features. BMC Health Services Research 2022;22:1526. https://doi.org/10.1186/s12913-022-08783-9.

[64] World Health Organization. WHO recommendations: non-clinical interventions to reduce unnecessary caesarean sections. World Health Organization; 2018.

[65] Chauhan BF, Jeyaraman M, Mann AS, Lys J, Skidmore B, Sibley KM, et al. Behavior change interventions and policies influencing primary healthcare professionals' practice—an overview of reviews. Implementation Science 2017;12:3. https://doi.org/10.1186/s13012-016-0538-8.

[66] Zahroh RI, Sutcliffe K, Kneale D, Vazquez Corona M, Betrán AP, Opiyo N, et al. Educational interventions targeting pregnant women to optimise the use of caesarean section: What are the essential elements? A qualitative comparative analysis. BMC Public Health 2023;23:1851. https://doi.org/10.1186/s12889-023-16718-0.

[67] Geerligs L, Rankin NM, Shepherd HL, Butow P. Hospital-based interventions: a systematic review of staff-reported barriers and facilitators to implementation processes. Implementation Science 2018;13:36. https://doi.org/10.1186/s13012-018-0726-9.

[68] Kingdon C, Downe S, Betran AP. Women's and communities' views of targeted educational interventions to reduce unnecessary caesarean section: a qualitative evidence synthesis. Reproductive Health 2018;15:130. https://doi.org/10.1186/s12978-018-0570-z.

[69] Atkins L, Francis J, Islam R, O'Connor D, Patey A, Ivers N, et al. A guide to using the Theoretical Domains Framework of behaviour change to investigate implementation problems. Implement Sci 2017;12:77. https://doi.org/10.1186/s13012-017-0605-9.

[70] Michie S, van Stralen MM, West R. The behaviour change wheel: A new method for characterising and designing behaviour change interventions. Implement Sci 2011;6:42. https://doi.org/10.1186/1748-5908-6-42.

AVAILABILITY OF DATA AND MATERIALS

This study is based on published articles, all of which are either publicly available or accessible through academic institutional credentials. The authors confirm that all other relevant data are included within the main text and/or additional files.

ABBREVIATIONS

- CS: Caesarean section
- DRG: Diagnosis-Related Group
- GRADE: Grading of Recommendations Assessment, Development and Evaluation
- OR: Odds ratio
- PRISMA-ScR: Preferred Reporting Items for Systematic Reviews and Meta-Analyses extension for Scoping Reviews
- RoB 2: Cochrane Risk of Bias tool
- RR: Risk ratio
- WHO: World Health Organization
- WMD: Weighted mean difference

ETHICAL APPROVAL

The data for this study consisted of published articles and therefore ethics approval was not needed.

FUNDING

This work received funding from the UNDP-UNFPA-UNICEF-WHO-World Bank Special Programme of Research, Development and Research Training in Human Reproduction (HRP), a cosponsored programme executed by the World Health Organization. MAB's time is supported by an Australian National Health and Medical Research Council (NHMRC) Investigator grant (2025634). The views expressed in this article are those of the authors and do not necessarily reflect the funders.

CONFLICTS OF INTEREST

No competing interests.

CONTRIBUTORSHIP STATEMENT

AH, APB, FD, MAB, MVC, NM, NO, RIZ, TMS, and VG contributed to the conceptualisation and study design. APB, DK, MAB, and VG secured funding. AH, MVC, and RIZ conducted data collection. AH, MVC, NM, and RIZ carried out data curation. AH, APB, FD, MAB, MVC, RIZ, TMS, and VG contributed to investigation, methodology, and formal analysis. RIZ prepared the original draft of the manuscript. All authors contributed to the review and editing of the final manuscript.

SUPPORTING INFORMATION FILES

- S1 Appendix. Preferred Reporting Items for Systematic reviews and Meta-Analyses extension for Scoping Reviews (PRISMA-ScR) Checklist.
- S2 Appendix. Type of interventions, mapped to the WHO health system building blocks.
- S3 Appendix. Search strategy.
- S4 Appendix. Critical appraisal.
- S5 Appendix. Outcomes and impact reported for each study.
- S6 Appendix. Table of included study characteristics.

Figure 1

Click here to access/download;Figure;Fig1.tif

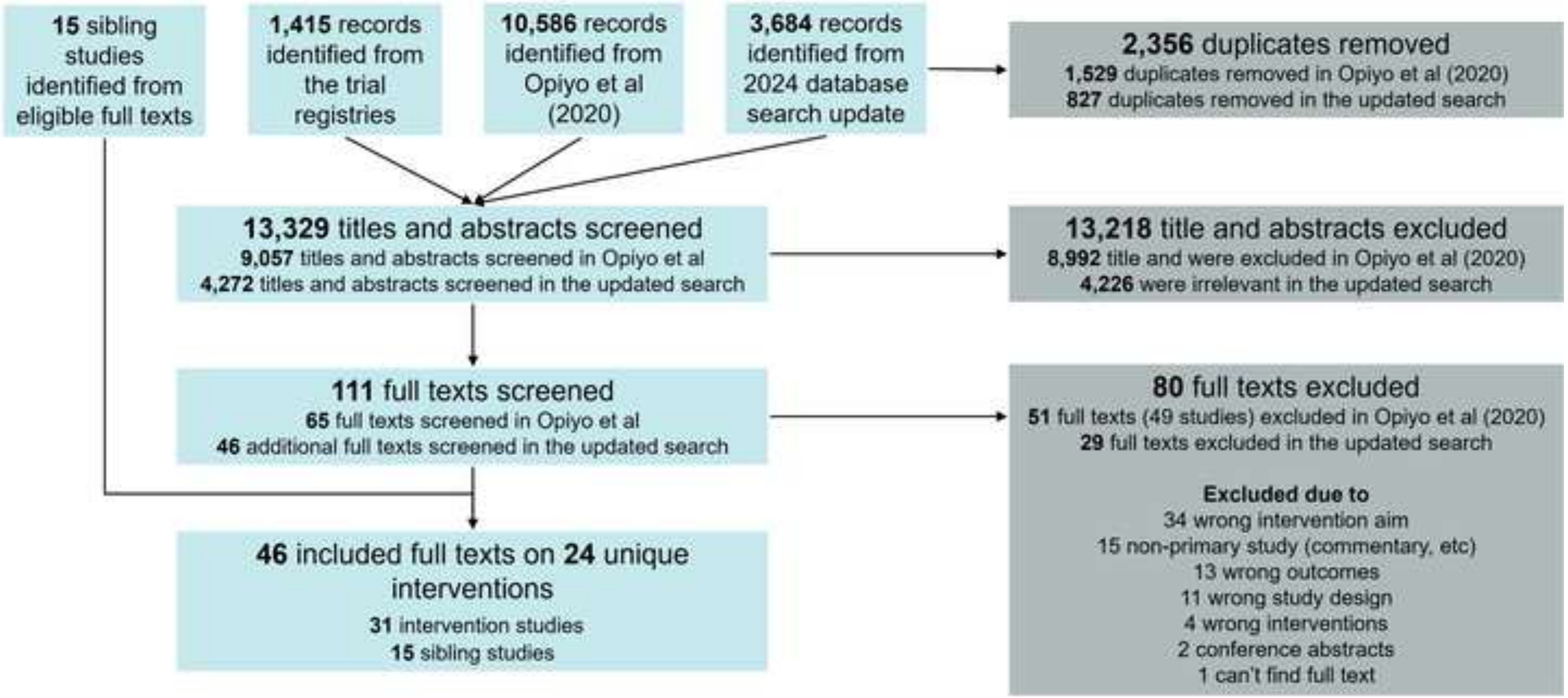

Figure 2

Click here to access/download;Figure;Fig2.tif

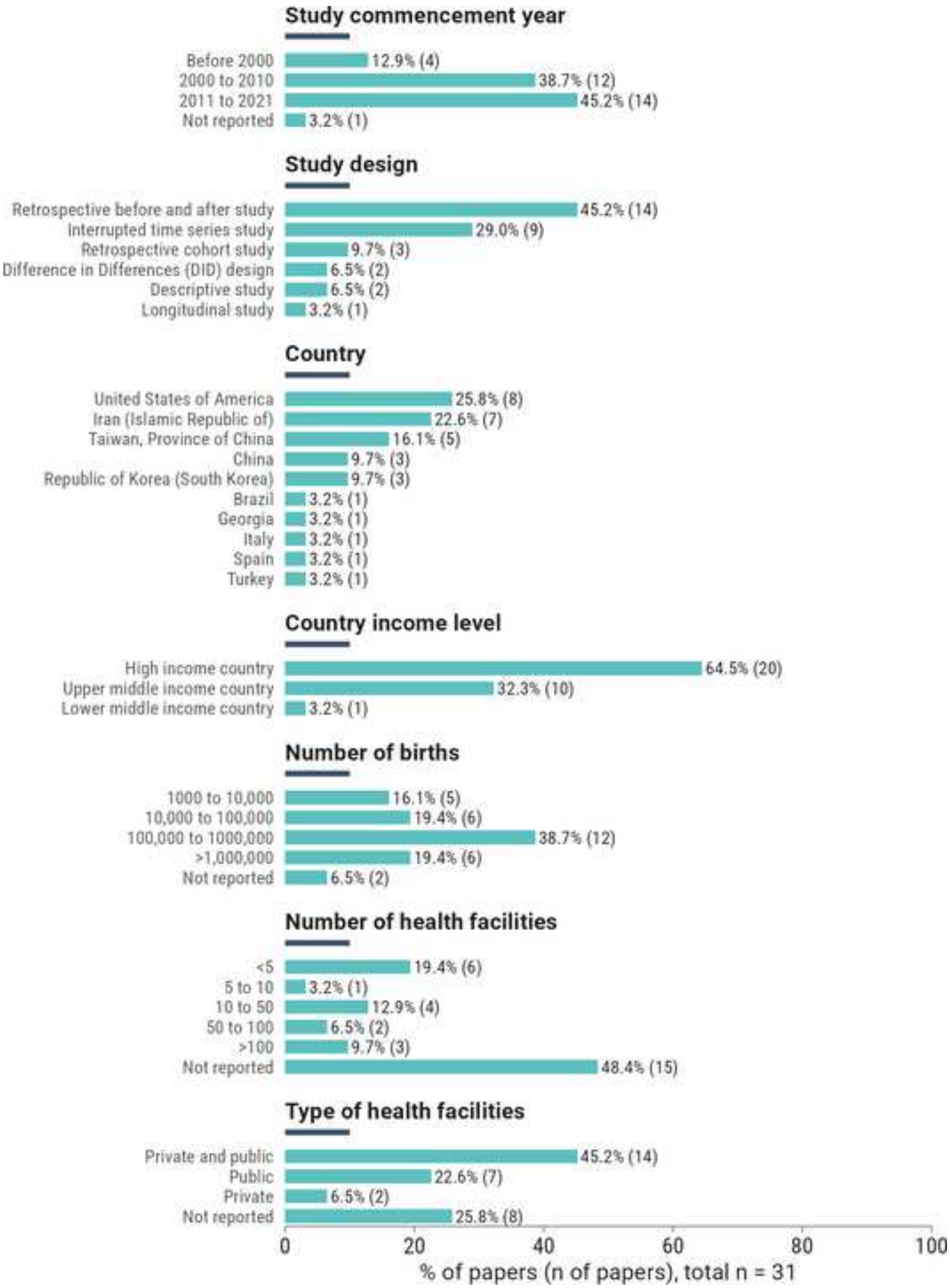

Figure 3

Click here to access/download;Figure;Fig3.tif

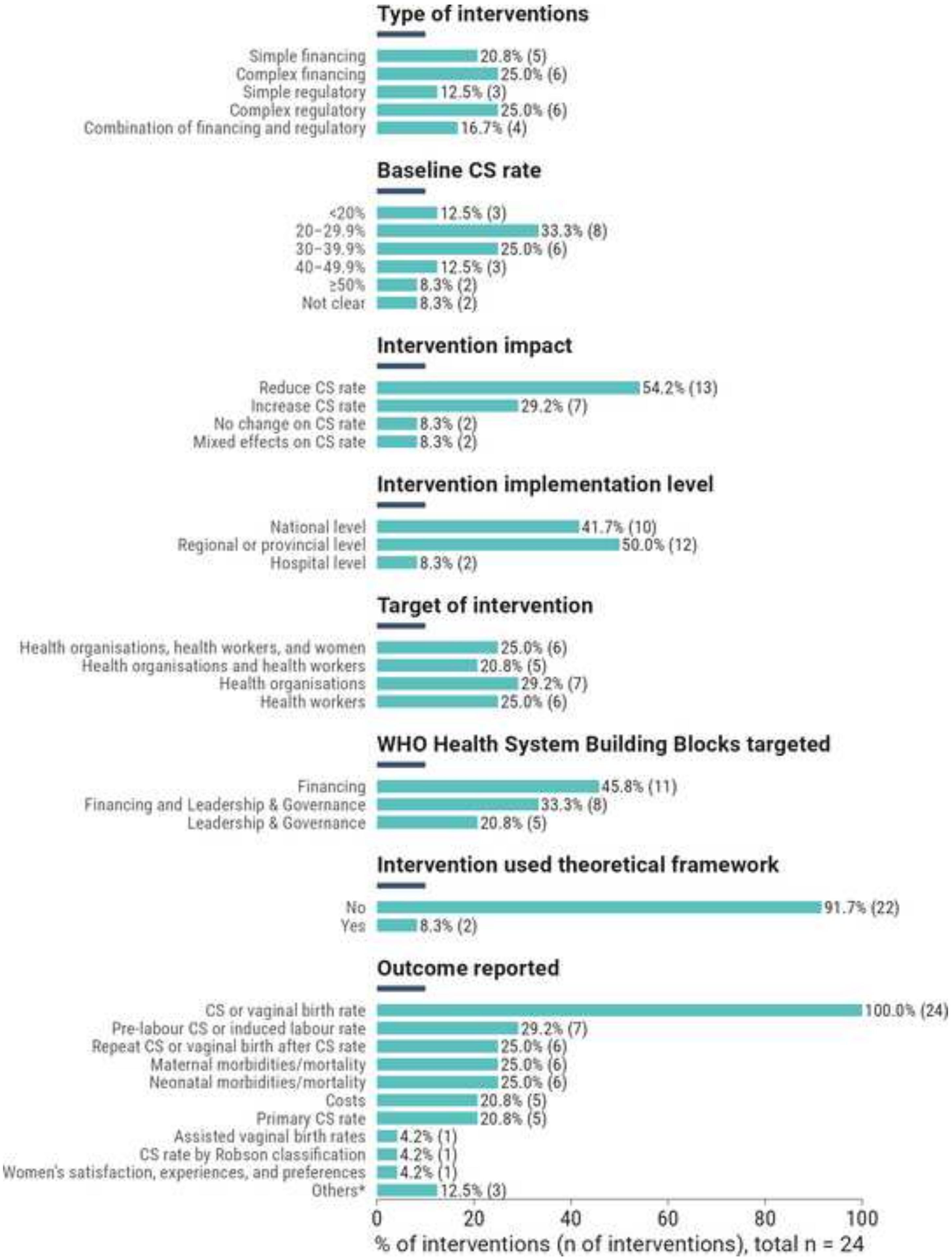

Figure 4

Click here to access/download;Figure;Fig4.tif

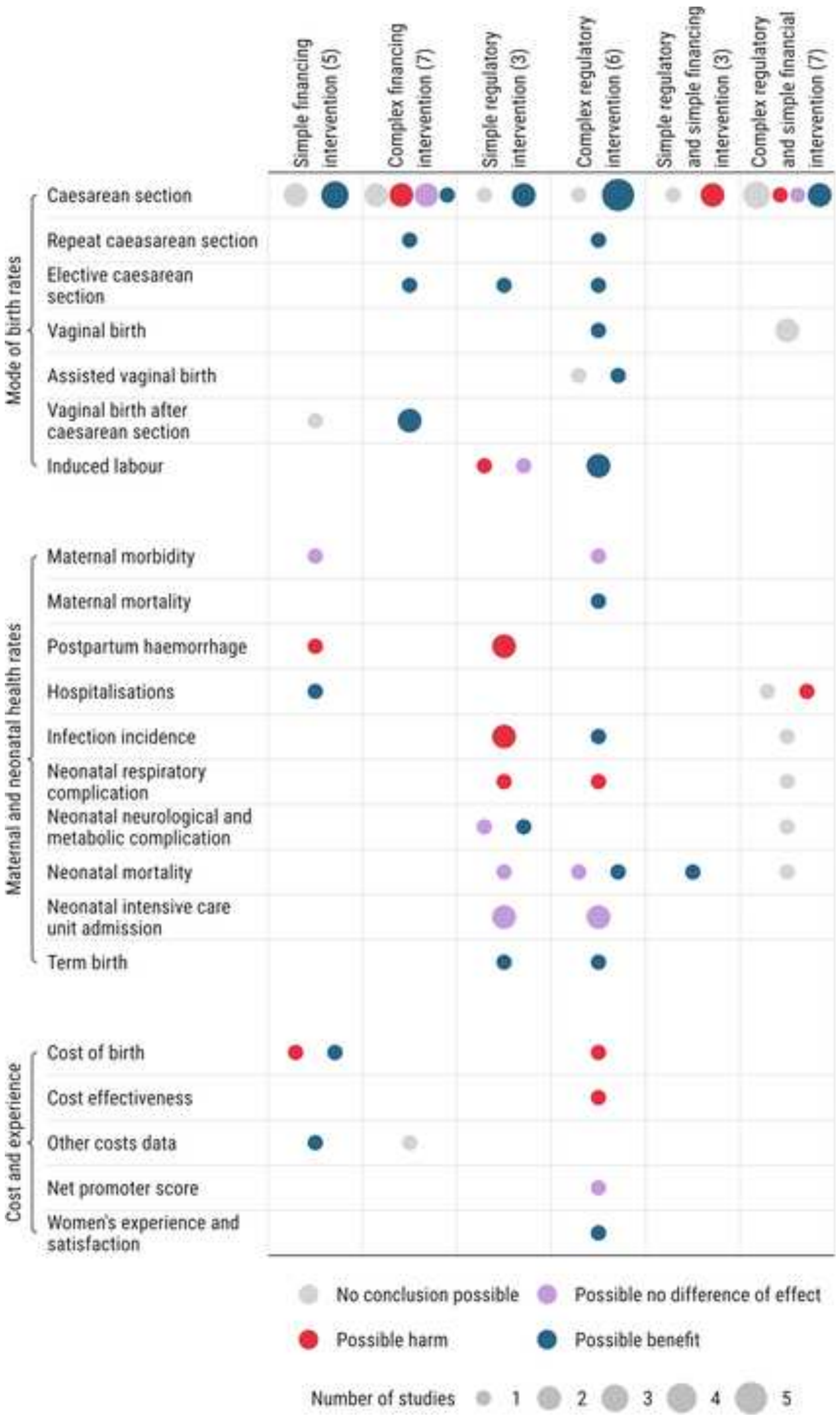

Click here to access/download
Supporting Information
S1 Appendix_28072025.docx

S2 Appendix

Click here to access/download
Supporting Information
S2 Appendix_28072025.docx

S3 Appendix

Click here to access/download
Supporting Information
S3 Appendix_28072025.docx

Click here to access/download
Supporting Information
S4 Appendix_28072025.docx

Click here to access/download
Supporting Information
S5 Appendix_28072025.docx

Click here to access/download
Supporting Information
S6 Appendix_28072025.docx
