# Supplementary material for: Financial and regulatory interventions to reduce unnecessary caesarean sections: An updated scoping review
Source: PLOS Glob Public Health. 2026 Feb 9;6(2):e0005830. doi: 10.1371/journal.pgph.0005830 (PMC12885279; doi:10.1371/journal.pgph.0005830)
Supplement: Attachment — Submitted filename: 20251024_Response to reviewers.pdf [file pgph.0005830.s011.pdf]

Feedback	Response to feedback
**Journal requirements**	
1. Please provide a detailed online Financial Disclosure statement. This is published with the article. It must therefore be completed in full sentences and contain the exact wording you wish to be published.<br><br>a) Please clarify all sources of financial support for your study. List the grants, grant numbers, and organizations that funded your study, including funding received from your institution. Please note that suppliers of material support, including research materials, should be recognized in the Acknowledgements section rather than in the Financial Disclosure.<br><br>b) State the initials, alongside each funding source, of each author to receive each grant. For example: "This work was supported by the National Institutes of Health (####### to AM; ###### to CJ) and the National Science Foundation (###### to AM)."<br><br>c) State what role the funders took in the study. If the funders had no role in your study, please state: "The funders had no role in study design, data collection and analysis, decision to publish, or preparation of the manuscript."	Thank you, we have provided these details at the end of the manuscript and on the journal submission platform.
2. Please ensure that the funders and grant numbers match between the Financial Disclosure field and the Funding Information tab in your submission form. Note that the funders must be provided in the same order in both places as well.	Thank you, we have ensured consistency in the funders, grant numbers, and their ordering.
3. Please update your online Competing Interests statement. If you have no competing interests to declare, please state: "The authors have declared that no competing interests exist."	Thank you. We have added a Competing Interests Statement: "The authors have declared that no competing interests exist."
4. If the reviewer comments include a recommendation to cite specific previously published works, please review and evaluate these publications to determine whether they are relevant and should be cited. There is no requirement to cite	Thank you. No reviewers suggested any citations to be included.

these works unless the editor has indicated otherwise.	
5. Please review your reference list to ensure that it is complete and correct. If you have cited papers that have been retracted, please include the rationale for doing so in the manuscript text, or remove these references and replace them with relevant current references. Any changes to the reference list should be mentioned in the rebuttal letter that accompanies your revised manuscript. If you need to cite a retracted article, indicate the article's retracted status in the References list and also include a citation and full reference for the retraction notice.	Thank you. We have checked the accuracy of our reference list.
The manuscript is extremely relevant and deals with an interesting theme. There are however some comments that we share with you from multiple reviewers. As you can see, we could gather interest from 6 reviewers for your manuscript, which is indicative of the relevance of your manuscript. We admit that there might be some overlap and perhaps even some minor conflicts in the recommendation, but request you to address them in the tabular response to comments document. We are confident that addressing these comments will improve the quality of your manuscript significantly.	Thank you for the kind comment, we have responded suggestions in the table as requested.
**Editor's feedback**	
**Summary and Overall Impression**<br><br>This manuscript provides an updated scoping review of financial and regulatory interventions to reduce unnecessary caesarean sections (CS). The authors identify 46 papers (31 interventions, 15 siblings) covering 24 unique interventions, and conclude that complex regulatory strategies combining guidelines, thresholds, and penalties/incentives show more consistent benefits than simple financial or regulatory levers. The topic is highly relevant and policy-significant, and the manuscript is well structured, registered, and PRISMA-ScR compliant. Its strengths include a clear intervention typology, inclusion of sibling studies, and acknowledgement of unintended harms. However, there are several	Thank you for the kind comment and suggestion.

methodological and reporting issues that should be corrected before the paper can be considered for publication. I recommend major revisions.	
**Major Issues (with page/line references)**<br><br>**1) Risk-of-bias tool misspecification**<br>*Page 7, lines 168–176*: The manuscript states that the **Cochrane RoB 2** tool was used, yet the domains listed (confounding, participant selection, classification of interventions, deviations, missing data, outcome measurement, reporting) correspond to **ROBINS-I** for non-randomised studies. Please clarify which tool(s) were used by study design (RoB 2 for RCTs, ROBINS-I for observational studies) and relabel figures/tables accordingly.	Thank you for flagging this. We used ROBINS-I but wrote it incorrectly in the manuscript. We have now edited, which you can find in Line 187-188 page 5:<br><br>*The Cochrane Risk of Bias In Non-Randomized Studies of Interventions (ROBINS-I) tool was used to appraise the quality of the included studies.*
**2) Eligibility criteria and time window**<br>*Page 6, lines 137–144*: The methods restrict inclusion to 2019–2024. *Page 11, lines 211–215*: However, several included evaluations are much older (e.g., Studnicki 1997, Tsai 2006, Lee 2007, Liu 2013). Please reconcile this inconsistency. Were older studies re-included as “siblings” or was the window relaxed? Update the PRISMA diagram and narrative to reflect this.	Thank you, this study only searched studies published from 2019-2024, as Opiyo et al have searched and included studies from journal inception to 2019. In this review, we have included and re-analysed the studies from the Opiyo et al also. That is why there are included studies published before 2019. We have tried to make this clearer by adding a sentence on Line 147-150, Page 5:<br><br>We used the search strategy from Opiyo et al (2020) [8] review for each database, and searched for new studies published between 1 January 2019 to 3 September 2024. *However, to get more comprehensive picture on the state of financial and regulatory interventions globally, our analysis also included studies from Opiyo et al (2020) review.*
**3) Definition of “low risk” populations**<br>*Page 5, lines 98–101*: “Low-risk women … with or without a previous CS (Robson groups 1–5).” Robson Group 5 is not generally considered “low risk.” Please clarify terminology and ensure consistent use of the Robson classification.	Thank you for this helpful comment. We agree that Robson Group 5 is not traditionally considered “low risk.” To address this, we have clarified our terminology in the revised text. We now define low-risk women as those in Robson Groups 1-4, while also noting that Group 5 was included because together Groups 1-5 represent the majority of women giving birth and are the main contributors to rising caesarean section rates globally. Please see Page 4, Line 105-109:<br><br>*Low-risk pregnant women were defined as those receiving antenatal and intrapartum care in health facilities, with term, singleton, and cephalic pregnancies, with or without a previous CS, corresponding to Robson groups 1 through 4 [24]. However, we also included any studies involving women in Group 5 (those with a previous caesarean section), as together Groups 1-5 represent the majority of*

	women giving birth and are the main contributors to the rising caesarean section rates globally [4].
**4) Strength of claims**<br>*Abstract, Page 2, lines 38–42*: "Complex regulatory interventions … revealed more benefits." *Discussion, Page 17, lines 365–373*: Similar over-statements of effectiveness. Please temper these claims to reflect the overall low/very-low certainty evidence base.	Thank you. We have revised this in the abstract:<br><br>*Complex regulatory interventions combining complex regulatory interventions that combined policy mandates with accountability mechanisms, health workers' training and guidance, incentives or penalties, women's engagement, and system-level coordination suggested potential benefit for birth outcomes compared with financing or simple regulatory interventions, though the certainty of evidence was low or very low, and such interventions may be costly.*<br><br>We have also tempered the language on effectiveness throughout, as seen in the manuscript. For example, under the intervention outcomes section, page 13 from Line 361 (see bolded text below):<br><br>Figure 4 shows the outcomes reported across different groups of interventions. Overall, studies evaluating complex regulatory interventions assessed a wider range of outcomes and **tended to report more possible benefits** related to mode of birth and maternal and neonatal health, compared with other intervention types. However, given the overall low to very low certainty of the evidence, these findings should be interpreted with caution. Among the six studies of complex regulatory interventions, several **reported possible reductions** in overall CS rate (5/6 studies; [49–53]), repeat CS (1/6 study; [52]), and elective CS (1/6 study; [49]), as well as **possible increases** in induced labour (2/6 studies; [50,53]), vaginal birth (1/6 study; [53]), and assisted vaginal birth rates (1/6 study; [50]). Some studies also **suggested possible benefits** for maternal and neonatal outcomes, including reduced maternal mortality (1/6 study; [53]), higher maternal satisfaction (1/6 study; [53]), lower infection rates (1/6 study; [53]), reduced neonatal mortality (1/6 study; [53]) and more babies born at term and with higher birth weights (1/6 study; [53]). **Possible harms** from increased neonatal respiratory complications were reported in one study [53]. Only one study examined cost outcomes, finding that the intervention was expensive and not cost-effective [53].
**5) Use of GRADE in a scoping review**<br>*Page 7, lines 173–178*: GRADE was applied to outcome certainty. This is unusual in scoping reviews. If retained, justify its use with methodological references and clarify it was interpretive only. Alternatively, move GRADE assessments to an appendix.	Although the use of GRADE is not typically required in scoping reviews, we applied it in an interpretive and exploratory manner to give readers a clearer understanding of the relative certainty of evidence across outcomes. This approach adds value by supporting a more nuanced interpretation of the mapped evidence.<br><br>Previous methodological work has noted that scoping reviews may incorporate elements of critical appraisal when it enhances the value of evidence mapping (Munn et al., 2018; https://doi.org/10.1186/s12874-018-0611-x). In our review, GRADE was used solely as a tool to guide interpretation rather than to formally rate, downgrade, or exclude evidence. We acknowledge its limitations in this

	context and have clarified that these assessments are descriptive in nature. For transparency, the full GRADE assessments are provided in the Appendix 5. And we have also added this rationale in the manuscript Page 6, Line 197-201:<br><br>*GRADE assessments are not standard practice in scoping reviews; however, consistent with methodological guidance that scoping reviews may include optional critical appraisal where it adds value [25], we applied GRADE in a descriptive and interpretive manner. The GRADE assessments were used only to contextualize and inform interpretation of the findings, not to exclude studies or formally rank the evidence. The results of the risk of bias and certainty assessments are presented in S5 Appendix.*
**6) Equity and generalisability**<br>*Results overview, Page 11, lines 218–220*: "The included studies were mostly conducted in high-income countries (20/31, 64.5%)." Please expand the Discussion to highlight the limited evidence from LMICs, where both CS overuse and underuse coexist, and reflect on transferability.	Thank you. We have added the following on the discussion, under implications for practice and research, Page 17-18, Line 485-489:<br><br>*More interventional research is needed in LMIC settings, as drivers of CS overuse and underuse may differ from high-income countries, which may limit the transferability of evidence from interventional research conducted in high-income countries. For example, limited surgical capacity, inequitable access, and variable clinical governance may influence the feasibility and impact of interventions.*
**7) Safety outcomes and unintended effects**<br>*Page 16, lines 361–364*: Simple regulatory interventions are linked to possible harms (postpartum haemorrhage, infection, neonatal complications). Please expand this section to specify which policies produced which harms (e.g., induction policies) and suggest safeguards (audit, VBAC readiness, staffing).	Thank you. We have added the simple regulatory interventions linked to possible harms on Page 16 Line 386-388:<br><br>*Two policies for induction of labour appeared to reduce CS rates but also showed potential harm: offering universal elective induction at 39 weeks gestation [46], and restricting elective induction before 39 weeks gestation [47].*<br><br>The suggestion on safeguards has already been covered in the discussion section on Page 17, Line 443-455.<br><br>*This review highlights the importance of anticipating and monitoring potential unintended effects on women's and babies' safety when implementing interventions to reduce CS rates. Several studies evaluating simple regulatory and simple financial interventions reported increases in adverse maternal outcomes (postpartum haemorrhage, maternal blood transfusion, chorioamnionitis) when CS was reduced [32,44,47]. These adverse outcomes may be attributable to longer or more complex labours as care shifts away from CS, particularly in cases of labour induction or attempts for vaginal birth in borderline or high-risk scenarios [32,44,47]. They may also arise from improved detection or reporting following changes in clinical protocols [32,44,45,47]. These findings highlight that a blunt reduction in overall CS rates, while appealing as a policy target, can be unsafe if it does not distinguish between medically necessary and unnecessary CS. Without adequate safeguards, such interventions risk inadvertently restricting access for women or babies with medically-necessary CS, rather than reducing unnecessary CS. A safe and equitable CS*

	reduction requires improved management of labour, audit and feedback mechanisms, and continuous monitoring to ensure that quality and timely access to medically indicated CS are not compromised [2].
**8) Cost and cost-effectiveness**<br>*Page 16, lines 343–348*: Only one complex regulatory study reported costs, finding the intervention not cost-effective. Please emphasise the paucity of cost data and recommend standardised reporting for future evaluations.	Thank you. We have included this in implications for practice and research, Page 17 Line 482-485:<br><br>*Evidence on the economic implications of regulatory strategies also remains limited. Only one complex regulatory intervention study reported cost data, and the intervention was not cost-effective. To inform policy and enable comparison across studies, future evaluations should include standardised and transparent reporting of costs and cost-effectiveness outcomes.*
**9) Minor Issues**<br>• Consistency in terminology: use "non-medically indicated/unnecessary CS" in line with WHO language (see Page 4, lines 53–54).	Thank you. As noted in the Background section, we use the term "unnecessary caesarean section" to refer to non-medically indicated caesarean section throughout the manuscript, for ease of reading and consistency. See Page 3, Line 56-59:<br><br>*Specifically, non-medically indicated CS (referred to as "unnecessary" CS) has been linked to not only adverse maternal outcomes, including severe morbidity, blood transfusion, hysterectomy, and ICU admission, but also neonatal outcomes, such as respiratory complications, NICU admission, and mortality [5,7].*
• Provide a supplementary intervention matrix mapping each intervention to WHO building blocks and "simple/complex" designations (refers to Table 1, Page 9, lines 196–195).	Thank you for this suggestion. To enhance clarity and transparency, we have added three additional columns on Appendix 7 to map each included intervention to the relevant WHO framework elements (as outlined in Table 1), its classification as simple or complex, and WHO health system building blocks.
• Clarify outcome categorisation labels ("possible benefit/no difference/harm") with examples (Page 10, lines 197–208).	Thank you. We have revised the text to include this explanation. Please see Page 7 and Line 222-230:<br><br>*We also categorised each reported outcome based on the classifications used in another review [31] to determine the level of impact of each intervention based on effect estimates and certainty of evidence. Outcomes were categorised as "possible benefit", "possible no difference of effect", or "possible harm" based on the reported effect estimates and confidence intervals. For example, a reduction in CS rates or improved maternal satisfaction was considered a "possible benefit", whereas no significant change in maternal mortality was classified as "possible no difference of effect". Interventions associated with increased complications, such as higher rates of postpartum haemorrhage, were classified as "possible harm". Table 2 summarises the outcomes, effect estimates, and certainty of evidence corresponding to each category. The type of outcomes and their impact classification for each study can be seen in S6 Appendix.*
• Ensure all figures (PRISMA, bubble plots) are included (Page 11, line 215 placeholder "[insert fig]").	Thank you. We have inserted all figures into the manuscript.
• Light editing to avoid repetition in Discussion	Thank you. We have revised the discussion section to minimise repetition and redundancy, please see in full of Page 16-18.

(Page 17–18, lines 365–388).	
• Contextualise findings against the *Lancet* CS Series (2018) and Cochrane review of non-clinical interventions.	Thank you. We have revised the discussion section to reflect this suggestion, please see in full of Page 16 Line 414-420:<br><br>*Our findings align with the Lancet CS Series (2018) [17] and the Cochrane review of non-clinical interventions [19], both of which emphasise that the determinants of CS use and the effectiveness of interventions are shaped by multi-level influences spanning women's preferences, health worker practices, institutional culture, and wider health system structures. Consistent with these studies, our review found that complex regulatory interventions, those combining multiple coordinated strategies across policy, facility, and community levels, were the ones that showed possible benefits in reducing CS rates.*
**Recommendation**<br>**Major revisions.** The manuscript addresses a critical global health question and has potential to make a meaningful contribution. However, methodological clarifications, tempering of claims, and fuller discussion of safety, cost, and generalisability are essential before it can meet the journal's standards.	Thank you. We have tried to address these points in our manuscript as explained above.
**Reviewer #1**	
Reviewer #1: Good work! A lot of effort went into this.	Thank you.
1. Please add date accessed for reference 2, 12 & add specific link to the file.	Thank you. We have added this.
2. This is a scoping review. I'm not sure why you would exclude studies aiming to increase CS use. While, we agree that unnecessary CS use should be avoided, it is a missed opportunity to critically evaluate the motivations behind centres who want to increase CS use. Knowledge translation is ineffective without understanding the opposing side of the story. (Line 122). If you want to argue significant knowledge addition since Opiyo et al (2020) was published, it would have been ideal to include those papers.	Thank you for this thoughtful comment. We agree that it is important, particularly in contexts where access is limited. However, our review was specifically designed to assess interventions aimed at reducing unnecessary CS use, which is a distinct policy and clinical context from interventions seeking to increase access. For example, strategies to expand caesarean provision in settings with underuse often focus on strengthening surgical capacity and improving access to emergency obstetric care, whereas strategies to reduce unnecessary caesarean in settings of overuse focus on regulatory, financial, or clinical governance mechanisms. To avoid conflating these very different contexts, we limited our scope to interventions targeting reduction of unnecessary caesarean. We have clarified this rationale in the Methods and acknowledge this as a limitation in the Discussion.<br><br>**Methods (Page 4, Line 130-133):**<br>*Interventions were eligible if they included financial and/or regulatory measures and were aimed at reducing unnecessary CS. We excluded studies aiming to increase CS use, because interventions intended to reduce unnecessary procedures reflect growing concern over the global oversupply of CS, associated health risks, and the strain on health systems [6,17].*

	**Discussion (Page 18, Line 511-514):**<br>*Lastly, this review did not include studies aimed at increasing CS use, which typically address barriers in under-resourced settings. While important, these strategies operate within a different policy and clinical context and were beyond the scope of this review.*
3. It's been a year since your last search strategy. You had almost 13k papers as such I will not advise an updated search at this time. However, this comment can't supersede a request for updated search by another reviewer.<br><br>Next time be more specific with your search to remove case reports and other exclusion studies upfront.	Thank you. We agree that the original search was extensive and comprehensive, identifying over 13,000 records across multiple databases. We have clarified in the Methods section that non-primary papers (e.g., case reports, editorials, reviews, and protocols; see Type of Studies section on Page 4 Line 101-102) were excluded during screening to improve specificity. Given the breadth and recency of the search and the inclusion of sibling and related studies, we believe the current evidence base remains up-to-date and sufficiently representative.
4. Next time when reviewing "sibling studies", please include both forward and backward citations. Not just forward citations. Use a tool like "Citation Chaser" to help you get the lists. Upload the list to Covidence afterwards.	Thank you. We acknowledge that bidirectional citation chasing (both forward and backward) is a valuable approach for future reviews, particularly where the evidence base is rapidly evolving. In this review, we conducted forward citation searching to identify subsequent related or updated studies linked to the original trials and evaluations, ensuring inclusion of the most recent evidence. Given the large number of records retrieved (over 13,000) and the comprehensive coverage of major databases and grey literature, we believe our approach was sufficient to capture all relevant sibling and related studies.<br><br>We appreciate your recommendation regarding the use of tools such as Citation Chaser and will consider incorporating these methods in future updates to further strengthen the search process.
5. You mention selection criteria for "sibling studies" (i.e., double check by another reviewer) but I do not see the exact screening criteria for main studies in the methodology. Please re-state it and don't assume the reader will be a scoping review expert to know this step.	Thank you. We have revised this section to make it clearer. Please see Page 5 Line 155-159:<br><br>We searched for sibling studies of the included interventions for additional information on the characteristics of the interventions or additional outcomes that may not have been presented in the papers retrieved by our searches. *Sibling studies were defined as publications linked to the same intervention as the main study but reported separately. These included, for example, formative research, process evaluations, costing analyses, and cost-effectiveness studies.*
6. Include the data extraction form in the appendix (if not included already) and mention it in the methods.	Thank you. We have added the data extraction form as an Appendix 4.
7. Next time use ROBINS-I for risk of bias analysis. RoB 2 is mostly for Clinical Trials but is acceptable here as a risk of bias is not necessary per se in scoping reviews. You didn't list the # of papers that evaluate policies. I'm not sure you can even do RoB 2 on those.	Thank you for flagging this. We used ROBINS-I but wrote it incorrectly in the manuscript. We have now edited, which you can find in Line 187-188, page 5:<br><br>*The Cochrane Risk of Bias In Non-Randomized Studies of Interventions (ROBINS-I) tool was used to appraise the quality of the included studies.*

	We also acknowledge that risk of bias assessment may not be strictly required in scoping reviews, but we included it to provide readers with a clearer understanding of the methodological quality across included studies.
8. I do not have the energy to open Opiyo et al (2020) to review the risk of bias for the remaining studies. Combine and include them here in your table appendix. Are you even serious with this comment: "The remaining study's quality appraisal can be seen on Opiyo et al (2020) review?." A very privileged statement expecting readers to find the access, time to download, review the other paper, whose first author is a co-author on this paper.	Thank you for this important feedback. We agree that it is the authors' responsibility to ensure readers have direct access to all relevant quality appraisal data within this review, without needing to consult previous publications. We have now integrated the risk of bias assessments from Opiyo et al. (2020) into Appendix 5, alongside the appraisals for the newly included studies.
9. It's very hard to read the results. You have regurgitated your data extraction sheet word for word. Can you make a few comparative statements between papers and improve the story a bit more with adding interpretation. Cut down the text in the result section by 10-25%. Please, this is supposed to be a journal article, not your dissertation :).	Thank you. We have revised the results section to reduce repetition and improve readability by incorporating comparative statements across studies and highlighting overall patterns and interpretations. Where possible, we have synthesised findings rather than listing individual study details verbatim, while retaining sufficient nuance for readers to understand intervention characteristics and outcomes.<br><br>Given the complexity of interventions and the diversity of contexts, some level of detail is necessary to ensure transparency and allow readers to interpret the results accurately. We believe the revised results strike a balance between conciseness and clarity.
10.This suggests that some interventions may lack the specificity or sensitivity needed to distinguish between necessary and unnecessary CS. Can you list all interventions that have a high vs low specificity and sensitivity so we get a take home message of what might work and what might not?	Thank you for this comment. Our understanding is that "specificity" and "sensitivity" are typically applied in the evaluation of diagnostic or screening tools. In our case, the interventions reviewed were complex health system interventions aimed at reducing caesarean section rates, rather than diagnostic tools. As such, assessing "specificity" and "sensitivity" is not directly applicable.<br><br>Instead, we focused on examining the direction and magnitude of effects, as well as identifying potential benefits and harms associated with each intervention type.
11. Cut down discussion by 10-25% and limit conclusion to one paragraph.	Thank you. We have reduced the discussion and conclusion throughout to make it more concise and to the point. Please see discussion and conclusion sections in the manuscript.
Reviewer #2	
Reviewer #2: The methodology of this work is well-documented and utilize a systematic approach and an exhaustive search process. The detailed systematic review of such a relevant topic (the impact of regulatory and financial interventions on the reduction of unnecessary cesarean) is highly valued.	Thank you.
This exploratory study contributes to the scientific literature by providing an updated review of the 2020 work	Thank you.

by Opiyo et al. It accurately demonstrates that while new studies have emerged, there has been no significant advancement in the quality of the evidence base, thus contributing to the reaffirmation of the need for additional scientific research, utilizing consistent scientific methodologies to generate high-quality evidence.	
It is recommended that the discussion of the results be cautious, given that it is based on studies with a low level of scientific evidence.	Thank you. We have tempered the language on effectiveness throughout, as seen in the manuscript. For example, under the intervention outcomes section, page 13 from Line 361 (see bolded text below):<br><br>Figure 4 shows the outcomes reported across different groups of interventions. Overall, studies evaluating complex regulatory interventions assessed a wider range of outcomes and **tended to report more possible benefits** related to mode of birth and maternal and neonatal health, compared with other intervention types. However, given the overall low to very low certainty of the evidence, these findings should be interpreted with caution. Among the six studies of complex regulatory interventions, several **reported possible reductions** in overall CS rate (5/6 studies; [49–53]), repeat CS (1/6 study; [52]), and elective CS (1/6 study; [49]), as well as **possible increases** in induced labour (2/6 studies; [50,53]), vaginal birth (1/6 study; [53]), and assisted vaginal birth rates (1/6 study; [50]). Some studies also **suggested possible benefits** for maternal and neonatal outcomes, including reduced maternal mortality (1/6 study; [53]), higher maternal satisfaction (1/6 study; [53]), lower infection rates (1/6 study; [53]), reduced neonatal mortality (1/6 study; [53]) and more babies born at term and with higher birth weights (1/6 study; [53]). **Possible harms** from increased neonatal respiratory complications were reported in one study [53]. Only one study examined cost outcomes, finding that the intervention was expensive and not cost-effective [53].
The lack of robust scientific studies, particularly in low- and middle-income countries (where the largest increases in CS rates have been observed), limits the possibility of conducting a meta-analysis or presenting novel scientific findings. In this sense, the novel scientific contribution of the work may require more years with additional studies.	Thank you. We have added the following on the discussion, under implications for practice and research, Page 17-18, Line 485-489:<br><br>More interventional research is needed in LMIC settings, as drivers of CS overuse and underuse may differ from high-income countries, which may limit the transferability of evidence from interventional research conducted in high-income countries. For example, limited surgical capacity, inequitable access, and variable clinical governance may influence the feasibility and impact of interventions.
To strengthen the scientific contribution of the work, it is recommended that the author provides a rigorous justification and a detailed rationale for how the obtained results contribute to the current knowledge in the field.	Thank you. We have revised the first paragraph of discussion section to capture this:<br><br>*This review advances current knowledge by updating and expanding the evidence base since Opiyo et al. (2020), providing a more detailed typology of financial and regulatory interventions, highlighting emerging patterns of effectiveness and safety, and identifying persistent gaps in*

	cost-effectiveness, implementation, and evidence from low- and middle-income settings.
Reviewer #	
Reviewer #3: Your article is a timely and important update on financial and regulatory interventions to address the world's global cesarean delivery epidemic. It beautifully builds on and extends the 2020 scoping review by Opiyo et al., doubling the number of evaluations and providing a structured classification of interventions using WHO frameworks. Key strengths I identified include protocol registration, adherence to PRISMA-ScR, inclusion of sibling studies, and the balanced discussion of potential harms. Nonetheless, I recommend some key revisions to strengthen methodological clarity and also address some overstated conclusions. These include:	Thank you.
1) Please clarify and correct the mis-labelling of RoB tools (RoB 2 vs ROBINS-I), to address the risk of methodological bias (Page 7, lines 168–176.)	Thank you for flagging this. We used ROBINS-I but wrote it incorrectly in the manuscript. We have now edited, which you can find in Line 187-188, page 5:<br><br>*The Cochrane Risk of Bias In Non-Randomized Studies of Interventions (ROBINS-I) tool was used to appraise the quality of the included studies.*
2) Please clarify the inclusion of pre-2019 studies compared to the stated 2019–2024 window, and then update PRISMA accordingly (Page 6, lines 137–144; also Page 11, lines 211–215).	Thank you, this study only searched studies published from 2019-2024, as Opiyo et al have searched and included studies from journal inception to 2019. In this review, we have included and re-analysed the studies from the Opiyo et al also. That is why there are included studies published before 2019. We have tried to make this clearer by adding a sentence on Page Line 147-150:<br><br>We used the search strategy from Opiyo et al (2020) [8] review for each database, and searched for studies published between 1 January 2019 to 3 September 2024. *However, to get more comprehensive picture on the state of financial and regulatory interventions globally, our analysis also included studies from Opiyo et al (2020) review.*
3) In the Abstract and Discussion statements the paper seems to overstate the strength of its claims which do not align with the stated low/very-low certainty evidence (Page 2, lines 38–42; Page 17, lines 365–373.).	Thank you. We have revised this in the abstract:<br><br>*Complex regulatory interventions combining mandated guidelines, CS rate thresholds, and penalties or incentives showed signals of potential benefit for birth outcomes compared with financing or simple regulatory interventions, though the certainty of evidence was low or very low, and such interventions may be costly.*<br><br>We have also tempered the language on effectiveness throughout, as seen in the manuscript. For example, under the intervention outcomes section, page 13 from Line 361 (see bolded text below):

	Figure 4 shows the outcomes reported across different groups of interventions. Overall, studies evaluating complex regulatory interventions assessed a wider range of outcomes and **tended to report more possible benefits** related to mode of birth and maternal and neonatal health, compared with other intervention types. However, given the overall low to very low certainty of the evidence, these findings should be interpreted with caution. Among the six studies of complex regulatory interventions, several **reported possible reductions** in overall CS rate (5/6 studies; [49–53]), repeat CS (1/6 study; [52]), and elective CS (1/6 study; [49]), as well as **possible increases** in induced labour (2/6 studies; [50,53]), vaginal birth (1/6 study; [53]), and assisted vaginal birth rates (1/6 study; [50]). Some studies also **suggested possible benefits** for maternal and neonatal outcomes, including reduced maternal mortality (1/6 study; [53]), higher maternal satisfaction (1/6 study; [53]), lower infection rates (1/6 study; [53]), reduced neonatal mortality (1/6 study; [53]) and more babies born at term and with higher birth weights (1/6 study; [53]). **Possible harms** from increased neonatal respiratory complications were reported in one study [53]. Only one study examined cost outcomes, finding that the intervention was expensive and not cost-effective [53].
4) It is important to provide the article's rationale for applying GRADE in a scoping review (typically expected in a Systematic Reviews), or otherwise move this to the supplementary files section.	Although the use of GRADE is not typically required in scoping reviews, we applied it in an interpretive and exploratory manner to give readers a clearer understanding of the relative certainty of evidence across outcomes. This approach adds value by supporting a more nuanced interpretation of the mapped evidence.<br><br>Previous methodological work has noted that scoping reviews may incorporate elements of critical appraisal when it enhances the value of evidence mapping (Munn et al., 2018; https://doi.org/10.1186/s12874-018-0611-x). In our review, GRADE was used solely as a tool to guide interpretation rather than to formally rate, downgrade, or exclude evidence. We acknowledge its limitations in this context and have clarified that these assessments are descriptive in nature. For transparency, the full GRADE assessments are provided in the Appendix 5. And we have also added this rationale in the manuscript Page 6, Line 197-201:<br><br>*GRADE assessments are not standard practice in scoping reviews; however, consistent with methodological guidance that scoping reviews may include optional critical appraisal where it adds value [25], we applied GRADE in a descriptive and interpretive manner. The GRADE assessments were used only to contextualise and inform interpretation of the findings, not to exclude studies or formally rank the evidence. The results of the risk of bias and certainty assessments are presented in S5 Appendix*.
5) On key definitions for the study population (persons undergoing cesarean deliveries), please clarify the use of “low-risk” when including	Thank you for this helpful comment. We agree that Robson Group 5 is not traditionally considered “low risk.” To address this, we have clarified our terminology in the revised text. We now define low-risk women as those in

Robson Group 5 as this doe not seem to tie with the WHO Robson framework definitions (Page 5, lines 98–101.).	Robson Groups 1-4, while also noting that Group 5 was included because together Groups 1-5 represent the majority of women giving birth and are the main contributors to rising caesarean section rates globally. Please see Page 4, Line 105-109:<br><br>*Low-risk pregnant women were defined as those receiving antenatal and intrapartum care in health facilities, with term, singleton, and cephalic pregnancies, with or without a previous CS, corresponding to Robson groups 1 through 4 [24]. However, we also included any studies involving women in Group 5 (those with a previous caesarean section), as together Groups 1-5 represent the majority of women giving birth and are the main contributors to the rising caesarean section rates globally [4].*
6) I propose to adopt an equity and generalisability lens to expand discussion on the implications for LMICs, where finances and regulatory interventions could underlie the coexistence of CS underuse and overuse.	Thank you. We have added the following on the discussion, under implications for practice and research, Page 17-18, Line 485-489:<br><br>*More interventional research is needed in LMIC settings, as drivers of CS overuse and underuse may differ from high-income countries, which may limit the transferability of evidence from interventional research conducted in high-income countries. For example, limited surgical capacity, inequitable access, and variable clinical governance may influence the feasibility and impact of interventions.*
7) The article could highlight the lack of cost-effectiveness evidence and recommend systematic cost reporting in future evaluations.	Thank you. We have included this in implications for practice and research, Page 17 Line 482-485:<br><br>*Evidence on the economic implications of regulatory strategies also remains limited. Only one complex regulatory intervention study reported cost data, and the intervention was not cost-effective. To inform policy and enable comparison across studies, future evaluations should include standardised and transparent reporting of costs and cost-effectiveness outcomes.*
Other minor improvements include addressing some redundancy in the Discussion section.	Thank you. We have revised the discussion section to minimise repetition and redundancy, please see in full of Page 16-18.
Overall, with these revisions, this paper has the potential to make a valuable contribution to policy and practice in global maternal health.	Thank you.
Reviewer #4	
Reviewer #4: 1. In Table 1, consider adding examples from included studies to illustrate categories (e.g., link to specific interventions) for better understanding. Also, the categories in this table would be more useful if the results are aligned with them.	Thank you. We did not use the intervention types in Table 1 as the primary structure for presenting the results because many interventions can include multiple elements in one of their interventions. For example, a case-based payment reform implemented only for publicly insured women could fall under both payment methods and provider selection processes. Instead, we used the framework in Table 1 as a conceptual guide to distinguish simple from complex interventions. Interventions were classified as simple when they involved a single element (e.g., shifting from fee-for-service to a global budget, representing one payment method change), and complex when they combined multiple elements within a single intervention (e.g., laws

	enforcing clinical guidelines, hospital review boards, and mandatory reporting). These complex interventions drew on several elements outlined in Table 1, including policy formulation, quality improvement, and accountability. The results were therefore conceptually aligned with Table 1 but presented according to the simple versus complex structure, which more accurately reflects the real-world overlap across intervention types within financial, regulatory, and combined interventions. Please see Page 6-7, Line 208-221.<br><br>*We also categorised interventions as either simple or complex based on adapted criteria from two WHO frameworks: 1) the WHO strategic health purchasing framework to classify health financing interventions [28], and 2) the WHO frameworks on health system governance for universal health coverage and primary healthcare operational to classify regulatory interventions [29,30]. These frameworks define different types of elements in financial and regulatory interventions, as outlined in Table 1. The framework in Table 1 was used as a guide for classifying interventions into simple or complex, recognising that a single intervention can include multiple elements (e.g., benefit design and payment methods).*<br><br>*Interventions were classified as complex if they implemented more than one type of element within a single intervention. For example, shifting from fee-for-service to a global budget was a "simple financing intervention" as it involved only a payment method change element per Table 1. In contrast, an intervention that combined laws to enforce clinical guidelines, hospital review boards, and mandatory reporting was a "complex regulatory intervention", as it involved several elements of regulatory interventions, per Table 1 (e.g., formulating policy and strategic plans, improving quality care, and ensuring accountability).*
2. Audits are described under Contracting modalities including reporting obligations as well as Ensuring accountability.	Thank you. As noted above, we used Table 1 as a reference to identify the types of elements present in financial and regulatory interventions, but did not assign each intervention rigidly to a single subcategory, since some intervention components, such as audits, can be relevant to multiple elements (e.g., both contracting modalities under reporting obligations and ensuring accountability). However, the framework was applied strictly to distinguish between simple and complex interventions. Interventions involving a single type of element were classified as simple, whereas those combining multiple elements within one program were classified as complex. We hope this is clearer.
3. Table 2 only presents terminology for low-certainty evidence, even though the inclusion criteria encompassed RCTs and quasi-experimental designs. As readers may expect at least some interventions to be evaluated in RCTs (which under GRADE would begin at high certainty), it would be important	Thank you for this helpful comment. Although our inclusion criteria allowed for RCTs and quasi-experimental designs, all included studies were observational or non-randomised. No RCTs were identified. According to GRADE guidance, randomised trials begin as high-certainty evidence, whereas non-randomised (observational) studies begin as low-certainty evidence and can only be upgraded under specific circumstances, such as when findings are consistent across multiple studies or demonstrate a large

for the authors to explain why the table is limited to low certainty as this is definition and not based on results.	effect size (https://www.jclinepi.com/article/S0895-4356(17)31031-4/abstract). Because each intervention in our review was reported in a single study, and meta-analysis was not feasible, certainty could not be upgraded. Therefore, the terminology in Table 2 is limited to "low" or "very low" certainty, consistent with established GRADE methodology. We have added footnote under Table 2 to explain this as well:<br><br>***All included studies were observational or non-randomised. Under the GRADE framework, observational studies begin as low-certainty evidence and can only be upgraded when findings are consistent across multiple studies or demonstrate very large effects [32,33]. As each intervention was reported in a single study, certainty could not be upgraded; therefore, all ratings presented here reflect low or very low certainty, consistent with GRADE guidance.*
4. Table 2 outlines standardised terminology for interpreting evidence certainty (e.g., 'may reduce,' 'may have no effect'), yet this phrasing is not consistently reflected in the Results or Discussion, where findings are reported narratively.	Thank you. We have revised and tempered the language on effectiveness throughout aligning with Table 2, as seen in the manuscript. For example, in the results section, Page 12-13, Line 320-335 (see bolded text below):<br><br>***COMPLEX REGULATORY INTERVENTIONS***<br>*Six studies evaluated the impact of complex regulatory interventions combining policy enforcement with multi-component quality improvement measures.* ***Possible benefits in reducing CS rates*** *were reported in studies from China [49], Georgia [50], USA [51,52], and Brazil [53], though the certainty of evidence was low. These initiatives implemented policy mandates (CS rate thresholds, signed informed consent requirements for maternal request by CS), accountability mechanisms (audit and feedback, public reporting), health worker support (training, guidelines), incentives or penalties (financial or recognition-based), women's engagement (birth plans, decision-making, prenatal education, birth companions), and system-level coordination (multi-stakeholder collaboration, integrated quality improvement). In contrast, one study in Spain [54], examining policy in promoting vaginal birth through updated protocols, training, infrastructure support, and women's involvement but reported an increase in CS rates.* ***The finding was inconclusive due to very low certainty of evidence****.*<br><br>*Overall, complex regulatory interventions that combined policy mandates with accountability mechanisms, workforce support, incentives or penalties, women's engagement, and system-level coordination* ***may have possible benefits in reducing CS rates,*** *whereas interventions focusing solely on service improvements without accountability mechanisms* ***may have no benefit or even increase CS rates****.*
5. The results are largely narrative. Consider including descriptive, study-level effect metrics such as absolute and relative changes in CS rates, reported RRs/ORs with 95% Cis etc,	Thank you for this helpful suggestion. We agree that including quantitative effectiveness metrics can be informative; however, the included studies reported caesarean section rates and changes using heterogeneous measures, for example, some used odds ratios or relative risks, others reported absolute percentage changes,

presented in tables or figures by intervention category.	several provided only narrative descriptions of change, some reported denominators, yet some did not. These outcomes were also measured across diverse contexts, populations, and time periods. As a result, it was not feasible to standardise or meaningfully compare effect metrics across studies without risking misinterpretation.<br><br>Instead, we focused on clearly communicating the direction of effect (reduction, increase, or no change), which is summarised in Appendix 7 and also categorised outcomes (possible benefit, possible harm, possible no difference in effect) in Appendix 6. This approach provides readers with a consistent and interpretable overview of each intervention's impact while preserving contextual nuance.<br><br>Given the multidisciplinary readership of this journal, including policymakers, programme implementers, district health planners, and hospital managers, we prioritised clarity and accessibility, ensuring that findings could be readily interpreted by readers from varied professional backgrounds. Those seeking detailed effect estimates can refer to the individual studies.
Reviewer #5	
SUMMARY OF FEEDBACK<br>This is a very interesting paper. Globally, there has been rise in caesarean section (CS)rates. Most of these rates are unnecessary and a cost on health systems. Interventions that can be implemented to reduce on the rates will tend to be welcome in most settings. Financial and regulatory interventions to reduce unnecessary caesarean sections have been implemented in a number of settings. However, most of the evidence on their effectiveness is scanty, inconclusive or not reliable.	Thank you.
The study has rightly pointed out reducing caesarean sections requires a combination of both financial and regulatory strategies. This is to ensure that there is a balance on approaches and avoid provider preferences if financial is considered only in absence of regulation.	Thank you
Abstract<br>Line 28-29. The first sentence under methods should ideally be last sentence under introduction. It gives reader an idea of the aim of the study.	Thank you. We have edited accordingly:<br><br>**Introduction:** Caesarean section (CS) is a life-saving procedure and critical component of comprehensive obstetric care. However, global CS rates are rising beyond levels justified by clinical indications. Growing evidence suggests health system supply-side factors, such as provider payment models that financially reward CS over vaginal birth and the absence of strict clinical guidelines, are contributing to this trend. Despite growing concerns, there is limited research assessing the implementation and impact of financial and/or regulatory interventions aimed at reducing unnecessary CS. *We conducted this scoping review to update, expand, and synthesise new evidence*

	published since a 2020 scoping review on financial and/or regulatory interventions.
Results:<br>Line 33. Did the 15 sibling studies included enhance scoping review of the subject? How did it do that?	Thank you for this comment. The 15 sibling studies enhanced the review by providing complementary information not reported in the primary papers. These included additional details on intervention design, implementation processes, contextual factors, and outcomes, such as cost, safety, and women's satisfaction, that were missing or only briefly described in the main publications. Including these sibling studies allowed for a more comprehensive and nuanced understanding of how the interventions were implemented and their broader effects across settings. This has also been explained in Methods section, Page 5 Line 155-164:<br><br>*We searched for sibling studies of the included interventions for additional information on the characteristics of the interventions or additional outcomes that may not have been presented in the papers retrieved by our searches. Sibling studies were defined as publications linked to the same intervention as the main study but reported separately. These included, for example, formative research, process evaluations, costing analyses, and cost-effectiveness studies. Sibling studies were those linked to the main intervention paper but published separately, such as formative research, process evaluations, costing, and cost-effectiveness studies.*
Introduction<br>I suggest that you consider defining what caesarean section rate is so that a reader can have clear understanding of the issue.	Thank you for this suggestion. Because this journal's readership largely comprises clinicians, researchers, and health system professionals familiar with the CS rate, we opted not to add a standalone definition. However, we have clarified the term briefly in the introduction to ensure accessibility for non-specialist readers, Page 4 Line 52-53:<br><br>*However, global rates of CS – the proportion of births by CS – are rising beyond levels justified by clinical need [3,4]*
Line 49-50. Consider providing the acceptable thresholds for CS, as guided by World Health Organization to appreciate statement that "global rates of CS are rising beyond levels justified by clinical need"	Thank you for this suggestion. The WHO does not recommend a specific acceptable threshold for caesarean section rates. Instead, WHO emphasises that CS should be performed only when medically indicated, as both very low and very high rates may indicate problems of access or overuse. Therefore, we have not added a specific threshold, as it would not reflect current WHO statement: https://www.who.int/publications/i/item/WHO-RHR-15.02
Line 54. Provide common examples of adverse maternal and neonatal outcomes linked with "unnecessary" CS	Thank you. We have added this on the introduction. See Page 3, Line 56-59:<br>*Specifically, non-medically indicated CS (referred to as "unnecessary" CS) has been linked to not only adverse maternal outcomes, including severe morbidity, blood transfusion, hysterectomy, and ICU admission, but also neonatal outcomes, such as respiratory complications, NICU admission, and mortality [5,7].*
Line 55. Do the supply-side health system factors happen to be drivers of high CS rates for both elective and non-elective cases or there is a distinction?	Thank you for this question. Supply-side health system factors can influence both elective and non-elective caesarean sections. However, their effects are often more pronounced in elective caesarean sections, as they strongly influence provider decision-making and practice

	patterns. As noted on Page 4, Lines 60-67, these factors include financial incentives within output-oriented payment systems that reward CS at higher rates than vaginal births; absence of robust audit systems or clear clinical guidelines; and medico-legal pressures that encourage CS as a defensive practice. In addition, competing demands, such as providers working across multiple facilities, may lead some to prefer CS because it is more predictable and easier to schedule and manage than vaginal birth.
Line 65-67. The sentence is quite interesting. WHO has noted that largest increase in CS rates is in low-and-middle-income countries? However, the study reports that most of the papers reviewed were from high-income countries. It would be nice if any of the reviewed papers was from the low-income countries to appreciate this statement.	Thank you for this insightful observation. We agree that the WHO has reported the most rapid increases in caesarean section rates occurring in low- and middle-income countries (LMICs). However, as noted in our results, the majority of studies meeting our inclusion criteria (20 of 31; 64.5%) were conducted in high-income countries. This likely reflects differences in research capacity, data availability, and policy documentation, rather than the absence of rising CS rates in LMICs. We have clarified this point in the Discussion, noting that while the trend is global, evidence on financial and regulatory interventions from LMICs remains limited and warrants further investigation, please see Page 17-18, Line 485-489:<br><br>*More interventional research is needed in LMIC settings, as drivers of CS overuse and underuse may differ from high-income countries, which may limit the transferability of evidence from interventional research conducted in high-income countries. For example, limited surgical capacity, inequitable access, and variable clinical governance may influence the feasibility and impact of interventions.*
Line 70-75. These citations are great. However, provide a summary of what they found.	Thank you. We have revised the paragraph to briefly summarise the main findings of each cited review, please see Page 5 Line 75-86:<br><br>*Several reviews have explored health system interventions aimed at reducing unnecessary CS. Chapman et al (2019) [18] and Chen et al (2018) [19] evaluated interventions targeting health workers and health organisations, such as audit and feedback, second opinion for CS decision, and peer review; both found inconsistent effects in reducing CS rates. Boatin et al (2018) [20] assessed audit and feedback interventions based on the Robson classification, reporting limited evidence of effectiveness. Torloni et al (2023) [21] assessed interventions to increase or reintroduce assisted vaginal birth (forceps or vacuum), highlighting feasibility challenges and the need for skill retention and training. Only two systematic reviews, by Yu et al (2019) [22] and Opiyo et al (2020) [8], evaluated both financial and regulatory interventions published up to 2018 and 2019, respectively; both found mixed effects and generally low-quality evidence [8,22]. More rigorous studies and innovative methods for assessing the impact of financial and regulatory interventions were deemed necessary on CS rates, safety, and cost-effectiveness [8,22]. In the five years since these previous reviews were published, new evidence may now be available to address these research gaps.*
Line 84-85. Consider providing clear explanation on the indicators to	Thank you for this comment. Our intention was to comprehensively map all outcomes reported in the included studies rather than restrict the review to

ascertain effectiveness, safety and outcomes for the study.	predefined indicators. All outcomes related to effectiveness (e.g., reductions in overall, elective, or repeat CS rates) and safety (e.g., maternal and neonatal morbidity, mortality, postpartum haemorrhage, infection, NICU admission, or respiratory complications) were extracted and summarised. This inclusive approach allowed us to capture the full range of outcomes reported across studies and intervention types. We have revised the text in the Methods to clarify this approach, see Page 7 Line 141-144:<br><br>*All outcomes related to the effectiveness (i.e., reduction of CS, repeat CS, and elective) or safety (i.e., maternal and neonatal health outcomes) of the intervention reported in the included studies were extracted, mapped, and reported.*
Line 132-134. Mention examples of indicators on effectiveness, safety and outcomes that you mapped. Be very clear.	Thank you for this comment. As above, examples of indicators for effectiveness and safety have already been provided in the Methods section under "Type of outcome measures" (e.g., reduction in overall, elective, or repeat CS; and maternal and neonatal health outcomes). Mapped outcomes are also presented in S6 Appendix and summarised in the Results section under "Intervention outcomes."
Line 139-141. What was the rationale for including papers excluded by Opiyo et al (2020)? By re-screening these papers, did you find something omitted that added to this current paper?	Thank you. As we aimed to include complex interventions involving financing and/or regulatory components, we re-screened all full-text papers excluded by Opiyo et al. (2020) to identify any studies that might meet our broader inclusion criteria. This ensured that multi-component or cross-cutting interventions, which may have been previously excluded, were captured to provide a more comprehensive understanding of the evidence base. This clarification has also been reviewed in the Methods section for clarity, see Page 5 Line 151-154:<br><br>*As we aimed to include complex interventions involving financing and/or regulatory measures, we re-screened all full-text papers excluded by Opiyo et al (2020) [8] to identify any studies that might meet our broader inclusion criteria.*
Line 152-159. Consider providing clear process used in including or excluding studies for review. How did the two authors choose which paper was to be included and was not to be included?	Thank you for this helpful comment. The process for study inclusion and exclusion has been clarified in the Methods section on "Selection of studies" sub-heading (page 5, Line 165-173):<br><br>*The titles and abstracts retrieved from various databases were compiled into a single reference database using EndNote (www.endnote.com), where duplicates were removed. Following deduplication, all references were imported into Covidence (www.covidence.org). We duplicated the Covidence project from the Opiyo et al (2020) [8] review, to not re-screen studies already included in their review. Two independent review authors assessed study eligibility based on the title and abstract against predefined inclusion and exclusion criteria. Studies meeting the inclusion criteria underwent full-text retrieval for further assessment. Studies that did not meet the inclusion criteria were excluded. In cases of disagreement*

	during any stage of screening and assessment, consensus was sought through discussion among the research team.<br><br>The selection criteria are detailed in methods section under “Criteria for considering studies for this review” sub-heading.
Line 166-167. How was testing for data extraction done? How was it reviewed by all the authors? How did we ensure that data extraction was sound and appropriate for the task?	Thank you. We have revised these details in the Methods section under “Data extraction” sub-heading for clarity, see Page 5 Line 178-185:<br><br>*We developed a specific form for data extraction for this review, including information on study objectives, methodology, design, settings, participant characteristics, and description of intervention (See Appendix 4 for data extraction details). The data extraction was pilot-tested by three reviewers on three studies to ensure accuracy and consistency, and reviewed by all authors to provide feedback before final use. One author conducted the primary data extraction, which was then independently cross-checked by another author. Any discrepancies were discussed and resolved through consensus with the wider author team. This process helped to ensure that the data extraction was both sound and appropriate for the review objectives.*
Line 218-221. How many included studies were from low-and-middle-income countries, if any? How was the picture on the indicators of interest on effectiveness, safety and outcomes from the low-and-middle-income countries compared to the high-income countries studies?	Thank you for this thoughtful comment. We acknowledge the importance of this question; however, there is no straightforward comparison possible given the contextual diversity and heterogeneity of the interventions included. While 11 of the 31 studies were conducted in LMICs, our review focused on mapping intervention types and outcomes rather than comparing results across income groups. We have already reported country-level context and corresponding intervention impacts in the Results section. Comparative analysis between LMICs and HICs would be more appropriate within a single intervention type (e.g., DRG implementation) rather than across the diverse set of interventions included here. This point has been noted in the Discussion as an area for future research (See Page 18, Line 509-511):<br><br>*This review did not compare intervention effects across low-, middle-, and high-income settings, as the included studies were highly heterogeneous in design, context, and intervention type.*
Line 233. Consider mentioning the theoretical and behaviour change frameworks used during intervention design for these two studies reviewed.	Thank you. We have added this on Page 14, Line 270-272:<br><br>*Only two interventions adopted theoretical or behaviour change frameworks during intervention design (Institute for Healthcare Improvement Scale-Up Framework and the Consolidated Framework for Implementation Research).*
Conclusion<br>The conclusion should reflect on the aim of the review and whether it has been achieved. Consider strengthening your conclusion based on great findings reported.	Thank you. We have revised the Conclusion to better reflect the aim of the review and to highlight how the findings address it. The updated conclusion is provided below:<br><br>*Despite the paucity of high-quality studies, current evidence allows for several important observations. Complex regulatory interventions that combine policy mandates with accountability mechanisms, health workers’*

	training and guidance, incentives or penalties, women's engagement, and system-level coordination tend to report greater possible benefits in reducing CS while maintaining safety, though they may be resource-intensive. Financial reforms that shift from fee-for-service to alternative payment models (e.g., DRGs) also showed more possible benefits in reducing CS rates than simply equalising fees between vaginal births and CS, yet evidence on safety remains limited. These regulatory and financial interventions must be supported by regulatory measures such as clinical audits to ensure the quality and safety. Unnecessary CS is influenced by both demand-side factors (e.g., women's preferences) and supply-side factors (e.g., provider behaviour, financial incentives, and regulatory frameworks). Because these drivers and system dynamics evolve over time, both financial and regulatory interventions must be embedded within an adaptive, agile health system that continuously monitors, evaluates, and adjusts policies to sustain safe, equitable, and effective care. Future research should focus on evaluating context-specific complex interventions that consider safety outcomes, women's choice, and employ rigorous and ethical study designs, and articulate the underlying theory of change to explain why effectiveness varies across settings.
#Reviewer 6	
Reviewer #6: 1. The manuscript is described as a scoping review, but parts of the analysis (e.g., GRADE certainty ratings) resemble a systematic review. The authors should clarify the intended scope and avoid overstating certainty.	Although the use of GRADE is not typically required in scoping reviews, we applied it in an interpretive and exploratory manner to give readers a clearer understanding of the relative certainty of evidence across outcomes. This approach adds value by supporting a more nuanced interpretation of the mapped evidence.<br><br>Previous methodological work has noted that scoping reviews may incorporate elements of critical appraisal when it enhances the value of evidence mapping (Munn et al., 2018; https://doi.org/10.1186/s12874-018-0611-x). In our review, GRADE was used solely as a tool to guide interpretation rather than to formally rate, downgrade, or exclude evidence. We acknowledge its limitations in this context and have clarified that these assessments are descriptive in nature. For transparency, the full GRADE assessments are provided in the Appendix 5. And we have also added this rationale in the manuscript Page 6, Line 197-201:<br><br>GRADE assessments are not standard practice in scoping reviews; however, consistent with methodological guidance that scoping reviews may include optional critical appraisal where it adds value [25], we applied GRADE in a descriptive and interpretive manner. The GRADE assessments were used only to contextualize and inform interpretation of the findings, not to exclude studies or formally rank the evidence. The results of the risk of bias and certainty assessments are presented in S5 Appendix.<br><br>We have also noted limitation on evidence certainty and temper language throughout.

2. Most interventions are from high-income countries, yet the fastest rise in CS rates is in LMICs. The discussion should more explicitly address how findings translate to LMIC settings and what gaps remain.	Thank you. We have added the following on the discussion, under implications for practice and research, Page 17-18, Line 485-489:<br><br>*More interventional research is needed in LMIC settings, as drivers of CS overuse and underuse may differ from high-income countries, which may limit the transferability of evidence from interventional research conducted in high-income countries. For example, limited surgical capacity, inequitable access, and variable clinical governance may influence the feasibility and impact of interventions.*
3. The results are detailed but sometimes difficult to follow. A concise summary table or figure comparing intervention type, setting, outcomes, and certainty of evidence would make the findings more accessible for policymakers and practitioners.	Thank you for this thoughtful suggestion. We agree that accessibility and clarity are important for policymakers and practitioners. While we did not add a new summary table, we have substantially revised the Results section to improve readability and flow, including clearer comparative statements across intervention types and streamlined descriptions of findings. The main characteristics, settings, outcomes, and certainty of evidence are already presented in Figure 2-4 and Appendices 5-7, which together provide a comprehensive and accessible overview of the results.
4. The manuscript notes adverse outcomes (e.g., postpartum haemorrhage, infection, neonatal respiratory issues) but does not explore mechanisms or propose mitigation strategies. A deeper analysis of how to prevent or monitor these harms is needed.	Thank you. We have expanded the Discussion to include a deeper analysis of potential mechanisms and mitigation strategies related to the adverse outcomes identified; See Page 17, Line 443-455:<br><br>*This review highlights the importance of anticipating and monitoring potential unintended effects on women's and babies' safety when implementing interventions to reduce CS rates. Several studies evaluating simple regulatory and simple financial interventions reported increases in adverse maternal outcomes (postpartum haemorrhage, maternal blood transfusion, chorioamnionitis) when CS was reduced [32,44,47]. These adverse outcomes may be attributable to longer or more complex labours as care shifts away from CS, particularly in cases of labour induction or attempts for vaginal birth in borderline or high-risk scenarios [32,44,47]. They may also arise from improved detection or reporting following changes in clinical protocols [32,44,45,47]. These findings highlight that a blunt reduction in overall CS rates, while appealing as a policy target, can be unsafe if it does not distinguish between medically necessary and unnecessary CS. Without adequate safeguards, such interventions risk inadvertently restricting access for women or babies with medically-necessary CS, rather than reducing unnecessary CS. A safe and equitable CS reduction requires improved management of labour, audit and feedback mechanisms, and continuous monitoring to ensure that quality and timely access to medically indicated CS are not compromised [2].*
5. While the policy implications are well stated, recommendations could be more specific on:<br>♣ Which types of interventions are most feasible in different health system contexts.<br>♣ How policymakers can balance financial reforms with regulatory safeguards.	Thank you. We appreciate the suggestion to provide more specific recommendations; however, assessing feasibility and prescribing implementation strategies were beyond the scope of this scoping review. Our aim was to map and synthesise the range and characteristics of financial and regulatory interventions, rather than to evaluate their contextual applicability or implementation feasibility.<br><br>Nonetheless, we have clarified the key patterns observed from the included studies. Specifically, we note that

♣ Strategies for engaging women and health workers in intervention design.	complex regulatory interventions combining policy mandates with accountability mechanisms, workforce support, incentives or penalties, women's engagement, and system-level coordination tended to report more possible benefits in reducing unnecessary CS and ensuring safety, albeit with low-certainty evidence and costly. Financial interventions involving structured payment reforms (e.g., DRG-based models) also showed possible reductions in CS rates, though results varied by context and lacked safety data.<br><br>We also highlight that engagement from key stakeholders' groups, such as women, health workers, policymakers, etc is important to influence intervention success. These have been added and clarified in the Implications for Practice and Research section (Page 28, Line 486-493).
