# Supplementary material for: Financial and regulatory interventions to reduce unnecessary caesarean sections: An updated scoping review
Source: PLOS Glob Public Health. 2026 Feb 9;6(2):e0005830. doi: 10.1371/journal.pgph.0005830 (PMC12885279; doi:10.1371/journal.pgph.0005830)
Supplement: Attachment — Submitted filename: Peer Review Report- PGPH-D-25-02141.docx [file pgph.0005830.s008.docx]

**Peer Review Report (line-specific)**

**PLOS Global Public Health: Financial and regulatory interventions to reduce unnecessary caesarean sections: an updated scoping review**

**Summary and Overall Impression**

This manuscript provides an updated scoping review of financial and regulatory interventions to reduce unnecessary caesarean sections (CS). The authors identify 46 papers (31 interventions, 15 siblings) covering 24 unique interventions, and conclude that complex regulatory strategies combining guidelines, thresholds, and penalties/incentives show more consistent benefits than simple financial or regulatory levers. The topic is highly relevant and policy-significant, and the manuscript is well structured, registered, and PRISMA-ScR compliant. Its strengths include a clear intervention typology, inclusion of sibling studies, and acknowledgement of unintended harms. However, there are several methodological and reporting issues that should be corrected before the paper can be considered for publication. I recommend major revisions.

**Major Issues (with page/line references)**

1) **Risk-of-bias tool misspecification**

*Page 7, lines 168–176*: The manuscript states that the **Cochrane RoB 2** tool was used, yet the domains listed (confounding, participant selection, classification of interventions, deviations, missing data, outcome measurement, reporting) correspond to **ROBINS-I** for non-randomised studies. Please clarify which tool(s) were used by study design (RoB 2 for RCTs, ROBINS-I for observational studies) and relabel figures/tables accordingly.

2) **Eligibility criteria and time window**

*Page 6, lines 137–144*: The methods restrict inclusion to 2019–2024. *Page 11, lines 211–215*: However, several included evaluations are much older (e.g., Studnicki 1997, Tsai 2006, Lee 2007, Liu 2013). Please reconcile this inconsistency. Were older studies re-included as “siblings” or was the window relaxed? Update the PRISMA diagram and narrative to reflect this.

3) **Definition of “low risk” populations**

*Page 5, lines 98–101*: “Low-risk women … with or without a previous CS (Robson groups 1–5).” Robson Group 5 is not generally considered “low risk.” Please clarify terminology and ensure consistent use of the Robson classification.

4) **Strength of claims**

*Abstract, Page 2, lines 38–42*: “Complex regulatory interventions … revealed more benefits.” *Discussion, Page 17, lines 365–373*: Similar over-statements of effectiveness. Please temper these claims to reflect the overall low/very-low certainty evidence base.

5) **Use of GRADE in a scoping review**

*Page 7, lines 173–178*: GRADE was applied to outcome certainty. This is unusual in scoping reviews. If retained, justify its use with methodological references and clarify it was interpretive only. Alternatively, move GRADE assessments to an appendix.

6) **Equity and generalisability**

*Results overview, Page 11, lines 218–220*: “The included studies were mostly conducted in high-income countries (20/31, 64.5%).” Please expand the Discussion to highlight the limited evidence from LMICs, where both CS overuse and underuse coexist, and reflect on transferability.

7) **Safety outcomes and unintended effects**

*Page 16, lines 361–364*: Simple regulatory interventions are linked to possible harms (postpartum haemorrhage, infection, neonatal complications). Please expand this section to specify which policies produced which harms (e.g., induction policies) and suggest safeguards (audit, VBAC readiness, staffing).

8) **Cost and cost-effectiveness**

*Page 16, lines 343–348*: Only one complex regulatory study reported costs, finding the intervention not cost-effective. Please emphasise the paucity of cost data and recommend standardised reporting for future evaluations.

9) **Minor Issues**

- Consistency in terminology: use “non-medically indicated/unnecessary CS” in line with WHO language (see Page 4, lines 53–54).
- Provide a supplementary intervention matrix mapping each intervention to WHO building blocks and “simple/complex” designations (refers to Table 1, Page 9, lines 196–195).
- Clarify outcome categorisation labels (“possible benefit/no difference/harm”) with examples (Page 10, lines 197–208).
- Ensure all figures (PRISMA, bubble plots) are included (Page 11, line 215 placeholder “[insert fig]”).
- Light editing to avoid repetition in Discussion (Page 17–18, lines 365–388).
- Contextualise findings against the *Lancet* CS Series (2018) and Cochrane review of non-clinical interventions.

**Recommendation**

**Major revisions.** The manuscript addresses a critical global health question and has potential to make a meaningful contribution. However, methodological clarifications, tempering of claims, and fuller discussion of safety, cost, and generalisability are essential before it can meet the journal’s standards.
