# Supplementary material for: Financial and regulatory interventions to reduce unnecessary caesarean sections: An updated scoping review
Source: PLOS Glob Public Health. 2026 Feb 9;6(2):e0005830. doi: 10.1371/journal.pgph.0005830 (PMC12885279; doi:10.1371/journal.pgph.0005830)
Supplement: Attachment — Submitted filename: 20251218_Response to reviewers.docx [file pgph.0005830.s012.docx]

| **Feedback from reviewers** | **Response to reviewers** |
| --- | --- |
| **Reviewer 4** |  |
| 1. The organization of Results by intervention type followed by simple/complex classification is appropriate, but operational details remain too minimal for readers to understand the practical content of interventions. Adding 1-2 sentences describing what was actually changed (payment rule, legal mechanism etc.) would improve interpretability and better reflect the aim of the scoping review. | Thank you for this helpful suggestion. We have revised the Results section to provide clearer operational descriptions of the interventions. Specifically, we have added 1-2 sentences at the start of each intervention category describing what was implemented/modified in practice (e.g., changes to payment structures, policy mandates, accountability mechanisms), to improve interpretability. |
| 2. The narrative reads like sequential study summaries rather than a mapped evidence landscape. Can add synthesized intervention outcome pattern statements. | Thank you for this helpful suggestion. To address this, we have revised the Results section to reduce sequential study-by-study descriptions and to more explicitly synthesise reported outcome patterns across studies within each intervention category. We now include summary statements that map the range of reported effects (e.g., possible benefit, possible no difference, possible harm) and highlight common features across interventions. |
| 3. Linking intervention descriptions more clearly to their intended mechanism for reducing unnecessary CS can add to the current structure | Thank you for this helpful suggestion. We have revised the Results section to more clearly link intervention components to their intended mechanisms for influencing caesarean section rates. For each intervention category, we now explicitly describe the mechanisms targeted (e.g., financial incentives, accountability, clinical decision constraints), while remaining descriptive and avoiding causal or effectiveness claims, in line with the purpose of a scoping review. |
| 4. Consider adding a summary table or infographic that clearly maps the intervention types and sub-types (24) alongside their reported direction of effect on caesarean-section rates, as this would greatly enhance clarity and allow readers to quickly grasp the evidence landscape. | Thank you for this helpful suggestion. We agree that a visual summary mapping intervention types and their reported direction of effect on caesarean section rates improves clarity.  In response, we would like to clarify that this mapping is already provided in **Figure 4**, which visually summarises intervention types alongside the reported direction of effect (possible benefit, possible no difference, possible harm, or no conclusion) across outcomes, with bubble size indicating the number of studies. In addition, **Appendix S6** presents a study-level summary of reported outcomes and effect classifications.  To improve visibility and reader guidance, we have revised the manuscript to more explicitly reference Figure 4 in the Results section and clarify its purpose in the figure caption. |
| **Reviewer #6** |  |
| The authors have address all the questions posed by the reviewers and hence can be considered for further process. | Thank you. |
